# An Optimized and Explainable Machine Learning Framework for Diabetes Prediction Using Marine Predators Algorithm and SHAP

**DOI:** 10.3390/jpm16080423

**Published:** 2026-08-10

**Authors:** Alifa Nasrin, Muhammad Bin Asif, Ramasamy Naidu, Afzal Haq Asif, Muhammad Shahzad Chohan, Gausal Azam Khan, Md Arifuzzaman, Akm Azad, Muhammad Ali Martuza

**Affiliations:** 1 Combined Military Hospital (CMH), Chattagram 4220, Bangladesh; 2 Clinical Department, Azerbaijani Medical University, Baku AZ1022, Azerbaijan; 3 Computer Engineering, CCSIT, King Faisal University, Al Ahsa 31982, Saudi Arabia; 4 Pharmacy Practice, College of Clinical Pharmacy, King Faisal University, Al-Ahsa 31982, Saudi Arabia; 5 Department of Clinical Nutrition, College of Applied Medical Sciences, King Faisal University, Al-Ahsa 31982, Saudi Arabia; 6 Civil and Environmental Engineering, College of Engineering, King Faisal University, Al-Ahsa 31982, Saudi Arabia; 7 Department of Mathematics and Statistics, Faculty of Science, Imam Mohammad Ibn Saud Islamic University (IMSIU), Riyadh 13318, Saudi Arabia; 8 Department of Computer Engineering, College of Computer, Qassim University, Buraydah 51452, Saudi Arabia

**Keywords:** diabetes prediction, machine learning, marine predators algorithm, XGBoost, SHAP, explainable artificial intelligence, SMOTE, hyperparameter optimization, clinical decision support, class imbalance

## Abstract

**Background:** Diabetes mellitus affects over 500 million people worldwide, yet many machine learning prediction models remain difficult to interpret, limiting their clinical applicability. This study proposes an explainable machine learning framework integrating the Marine Predators Algorithm (MPA) for hyperparameter optimization with SHAP-based explainability to diabetes prediction. **Methods:** Logistic Regression (LR), Random Forest (RF), and MPA-optimized XGBoost were evaluated using a publicly available Kaggle diabetes dataset of approximately 100,000 records. Statistical significance was assessed using Wilcoxon signed-rank tests with Bonferroni correction for fold-wise cross-validation results, while McNemar’s and DeLong’s tests were employed for paired comparison of independent test-set predictions and ROC-AUC values, respectively. Performance was assessed using accuracy, precision, recall, F1-score, specificity, ROC-AUC, and Brier score. SHAP was used to provide global and local model explanations. **Results:** The MPA-optimized XGBoost model achieved the highest performance, with 96.72% accuracy, 97.70% precision, 95.70% recall, 96.71% F1-score, and 99.56% ROC-AUC, significantly outperforming LR and RF (*p* < 0.001). The model demonstrated good calibration with a Brier score of 0.0262. SHAP analysis identified HbA1c level, blood glucose level, and age as the most influential predictors, while interaction analysis indicated a synergistic relationship between HbA1c and blood glucose. **Conclusions:** The proposed framework demonstrated strong predictive performance and interpretable model behavior on the publicly available diabetes dataset used in this study. These findings indicate the potential of MPA-based optimization combined with SHAP explainability for supporting transparent machine learning research in diabetes prediction. However, additional external validation using independent clinical datasets is required before considering the framework for clinical decision support or real-world deployment.

## 1. Introduction

A chronic metabolic disease that represents a serious danger to global health is diabetes mellitus, whose prevalence is increasing worldwide, attributed to sedentary lifestyles, poor diets, and aging populations [1]. Early diagnosis is crucial for preventing complications such as neuropathy, renal failure, and cardiovascular problems [2]. Machine learning (ML) techniques are increasingly employed for diabetes prediction by discovering hidden patterns in large healthcare datasets that are normally challenging to identify using conventional techniques [3]. Predictive modeling has become a key component in clinical decision support systems [4]; however, clinicians need explanations in addition to precise forecasts, necessitating explainable AI (XAI) in medical ML systems [5]. Diabetes mellitus is a chronic metabolic disorder characterized by elevated blood glucose levels resulting from impaired insulin secretion and insulin resistance [6]. Its development is strongly influenced by genetic, lifestyle, and environmental factors, and its prevalence is increasing globally due to sedentary behavior and unhealthy dietary habits [7]. It indicates that biological mechanisms such as gut microbiota imbalance and metabolic dysfunction, along with lipid metabolism regulation, play an important role in disease progression and management [8].

Biological, environmental, and lifestyle risk factors contribute to diabetes. Unhealthy diet is a major factor in insulin resistance, while inactivity contributes to obesity and metabolic dysregulation [9]. Hormonal regulation is also impacted by genetic, stress, and sleeping pattern irregularities [10]. Sensitivity to insulin decreases with age, and urbanization, processed foods, smoking, and alcohol consumption are strongly associated with type 2 diabetes [11]. Hypertension and cardiovascular disease complicate diagnosis, and limited access to healthcare results in detection delays, making diabetes prediction a complex multifactorial problem [12].

Several ML models are applied for diabetes prediction. Logistic Regression (LR) is preferred for interpretable results, while Support Vector Machines (SVMs) can handle nonlinear decision boundaries [13]. Decision Trees and Random Forests (RF) exploit complex feature interactions [14]. Gradient boosting algorithms perform well on tabular medical data and K-Nearest Neighbor (KNN) is an instance-specific approach [15,16]. Gradient boosting methods are accurate but computationally expensive and sensitive to hyperparameter settings [17]. Personalized medicine aims to tailor disease prevention, diagnosis, and therapeutic decision making according to individual patient characteristics rather than adopting a uniform clinical approach. In diabetes management, personalized risk assessment enables clinicians to identify individuals with distinct risk profiles and to provide timely, patient-specific interventions. Explainable machine learning further strengthens this objective by providing transparent explanations of model predictions, allowing clinicians to understand how demographic, clinical, and laboratory variables contribute to individualized diabetes assessment. Therefore, the proposed framework aligns with the principles of personalized medicine by supporting interpretable, patient-centered risk stratification and clinical decision support.

Although progress has recently been made, significant challenges remain, including limited explainability, weak calibration robustness, inadequate statistical validation, and lack of holistic comparison. Current approaches are also sensitive to class imbalance, hyperparameter tuning, and dataset bias, resulting in poor generalization. To address these problems, this work presents a comparative XML framework based on LR, RF, and MPA-optimized XGBoost, with SHAP-based interpretability, calibration analysis, and statistical validation to deliver accurate and to improve predictive performance, model interpretability, and methodological transparency for diabetes prediction under an experimental evaluation setting.

### 1.1. Research Questions and Hypotheses

In the identified research limitations of existing diabetes prediction models, this study aims to investigate whether integrating metaheuristic optimization with explainable machine learning can improve both predictive performance and model transparency. To guide the investigation, the following research questions are formulated:

- RQ1: Can Marine Predators Algorithm (MPA)-based hyperparameter optimization improve the predictive performance of XGBoost for diabetes classification compared with conventional machine learning models?
- RQ2: Does integrating SHAP-based explainable artificial intelligence improve the interpretability and transparency of diabetes prediction while maintaining high classification performance?
- RQ3: Does the proposed MPA-optimized XGBoost framework provide statistically reliable and well-calibrated predictions for diabetes classification?
- To answer these research questions, the following hypotheses are formulated:
- H1: The MPA-optimized XGBoost model achieves significantly higher predictive performance than baseline machine learning models.
- H2: SHAP-based explainability provides meaningful global and local interpretations of model predictions without reducing classification performance.
- H3: The proposed framework produces reliable and statistically significant prediction results, demonstrating robustness through calibration analysis, ablation studies, and statistical significance testing.

### 1.2. Key Contributions

The main contributions of this work are as follows:

- A comparative XML framework incorporating LR, RF, and XGBoost is developed to predict diabetes with high accuracy and clinical interpretability.
- A comprehensive preprocessing pipeline with missing-value imputation, categorical encoding, feature normalization, and class balancing is applied to enhance data quality and model performance.
- The Marine Predators Algorithm (MPA) is used to optimize hyperparameters of RF and XGBoost, enhancing classification performance, generalization ability, and robustness.
- SHAP-based explainability provides global and local interpretability for transparent and clinically meaningful diabetes risk investigation.
- The framework is validated using cross-validation, calibration analysis, ablation study, and statistical tests for robust and rigorous evaluation.

The remainder of this paper is organized as follows: Section 2 reviews previous methods for predicting diabetes. Section 3 presents the problem statement. Section 4 describes the proposed approach. Section 5 describes the experimental setup. Section 6 presents results and discussion. Section 7 concludes the paper and outlines future work.

## 2. Literature Review

Maimaitijiang et al. [18] introduced an explainable diabetes prediction model based on CatBoost, applying SHAP and SMOTE to increase predictive accuracy and explainability, together with an LLM-based chatbot for personalized suggestions. Nevertheless, self-reported information can be subject to bias, and without external validation, clinical applicability remains limited. A fuzzy-empowered ANN model for type 2 diabetes prediction with lifestyle calibration was designed by Ganie and Malik [19]; however, it is regionally biased. Netayawijit et al. [20] presented an interpretable ML framework based on SMOTE, SHAP, RF, XGBoost, and SVM, but it is primarily established on synthetic data and lacks real-world validation. Li et al. [21] proposed an iterative adaptive minimum spanning tree-based outlier detection method for medical datasets; however, its computational complexity may limit scalability in large-scale real-time applications. Mi et al. [22] investigated the ICAM1 rs5498 polymorphism and its association with diabetes mellitus risk through a meta-analysis; however, its findings are limited to genetic correlation and lack predictive modeling applicability. Chen et al. [23] proposed a dual-branch attention network combined with serum Raman spectroscopy for diabetic kidney disease diagnosis; however, the method focuses on post-complication detection rather than early-stage diabetes prediction.

Hoyos et al. [24] proposed a fusion approach based on statistical analysis, fuzzy clustering, ANN, SVM, and XGBoost, but it lacks robustness against uncertainty. Jasim et al. [25] proposed a hyper-optimized AdaBoost model based on grid search, but it is computationally intensive and less interpretable. Ashisha et al. [26] developed an IoMT-based framework using RF, GBM, LightGBM, and Decision Tree with Boruta and oversampling, but it does not address real-time or edge deployment.

Dharmarathne et al. [27] developed a self-explainable model via DT, KNN, SVC, and XGBoost using SHAP, but scalability to larger datasets is problematic. Rastogi et al. [28] proposed an IoT-based monitoring system with RF and physiological signals, but it focuses more on sensor integration than predictive robustness. Tasin et al. [29] combined XGBoost with SMOTE, ADASYN, LIME, and SHAP but did not perform calibration or uncertainty assessment.

Pang [30] used MARS and RF analysis with SHAP, but feature interaction and fairness analysis are not considered. Curia [31] introduced a multi-classifier scheme composed of DT, DNN, XGBoost, LR, KNN, and SVC with LIME, but it suffers from redundancy and overfitting. Iftikhar et al. [32] presented a deep learning model using SMOTE, SHAP, and LIME; however, it is computationally expensive and infeasible for resource-constrained environments. Tran et al. [33] proposed an explainable stacking ensemble framework incorporating feature tokenizer transformers for diabetes prediction in men, demonstrating that transformer-based feature representations combined with ensemble learning can improve predictive performance while maintaining interpretability. However, the proposed framework was designed for a specific population and did not investigate metaheuristic optimization techniques for model tuning. Islam et al. [34] developed an explainable machine learning framework that integrates hyperparameter tuning, SHAP analysis, partial dependence plots, and LIME explanations. Their results highlighted the effectiveness of combining multiple explainability methods to improve model transparency, although calibration analysis and statistical validation were not comprehensively evaluated.

Iftikhar et al. [35] investigated the integration of SMOTE with SHAP-based explainability to improve diabetes prediction under imbalanced class distributions. Their findings demonstrated improved classification performance and feature interpretation, but the optimization process relied on conventional tuning methods rather than swarm intelligence-based optimization. Rossi et al. [36] introduced the D.R.E.A.M. framework, which combined explainable AI techniques with ensemble learning for diabetes risk prediction. Although the framework achieved high predictive performance, the study primarily focused on explainability and did not include ablation analysis or comprehensive statistical significance testing. Ahmad et al. [37] proposed a stacked ensemble model enhanced with SHAP explainability for early-stage diabetes prediction. Their framework improved prediction accuracy and interpretability; however, external validation and advanced hyperparameter optimization strategies were identified as areas requiring further investigation. These studies demonstrate significant progress in developing explainable machine learning models for diabetes prediction. Nevertheless, important research gaps remain regarding the integration of metaheuristic hyperparameter optimization, comprehensive model calibration, statistical significance analysis, and systematic ablation studies within a unified prediction framework. To address these limitations, the proposed study combines Marine Predators Algorithm (MPA)-based hyperparameter optimization with XGBoost, SHAP explainability, calibration analysis, statistical validation, and ablation studies to develop a more robust and interpretable diabetes prediction framework.

In general, the prior literature demonstrates strong predictive performance but suffers from limitations including poor calibration, sensitivity to class imbalance, large computational demands, weak external validation, and limited clinical interpretability. In response, we propose a comparative XML framework integrating LR, RF, and MPA-XGBoost with SHAP interpretability, focusing on calibration reliability, statistical validation, and transparent explanations for clinically trustworthy diabetes prediction.

## 3. Problem Statement

Previous work on diabetes prediction employs statistical models such as LR, Bayesian networks, or survival analysis, which have limited predictive performance and cannot model nonlinear relations, resulting in low sensitivity in high-risk situations [38]. Rule-based systems apply fixed rules and are not adaptable to variations in patient status. Ensemble methods such as voting and bagging enhance stability, but generalization remains poor due to dataset bias and insufficient external validation [39].

Numerous deep learning models, including CNNs and RNNs, are being applied to medical data; however, their suitability for practical use is limited by the need for substantial computational resources and large datasets [40]. Hybrid optimization and swarm intelligence-based algorithms are employed for feature selection and hyperparameter tuning, which add complexity without improving interpretability. Graph-based and probabilistic models can represent patient relationships but suffer from scalability and real-world deployment challenges [41].

## 4. Proposed Approach

**Figure 1:**
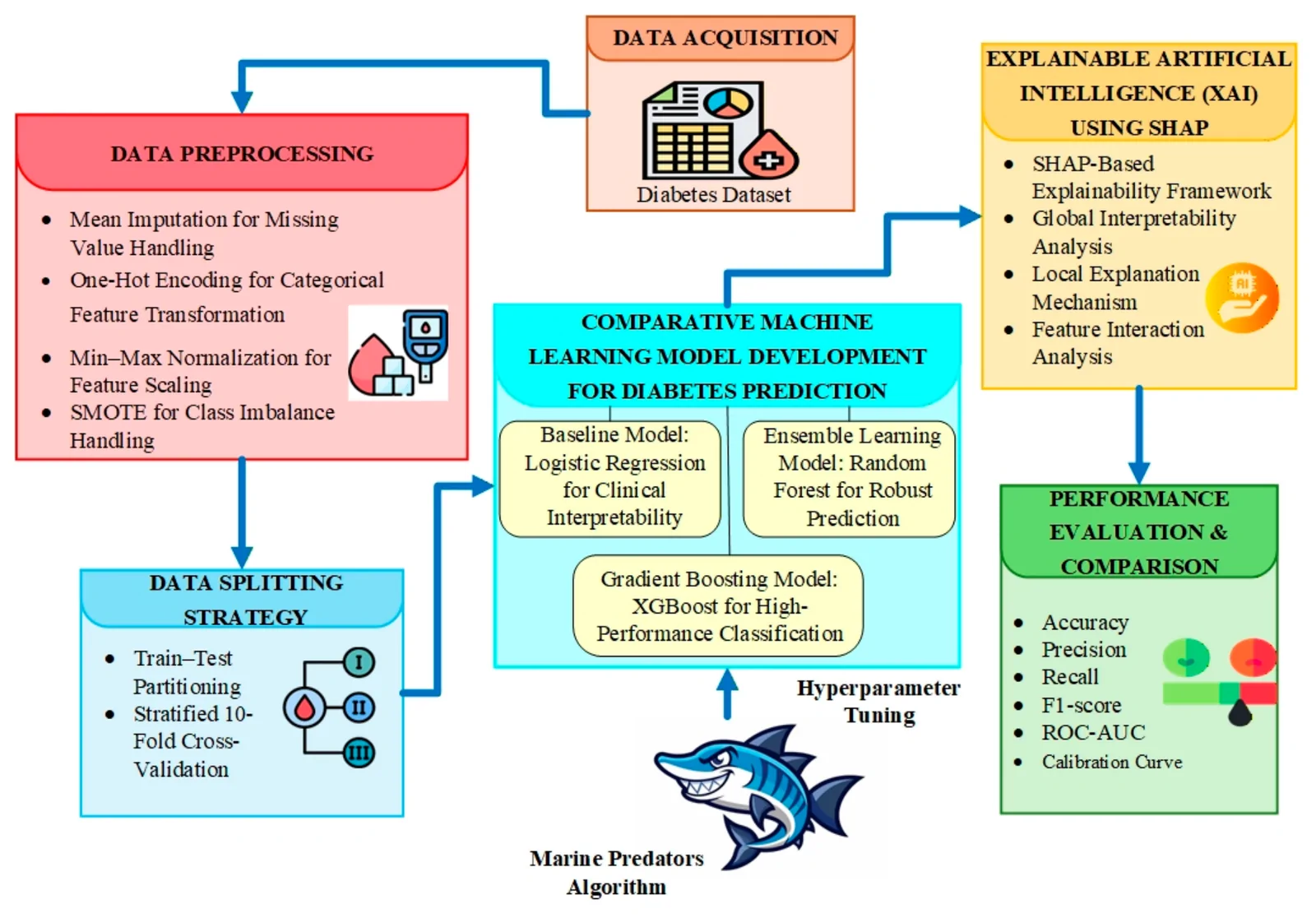
Block schematic of the integrated comparative and XAI framework.

The proposed diabetes prediction framework is illustrated in Figure 1, which summarizes an end-to-end pipeline from data collection and preprocessing to model building, tuning, and testing. The system starts with dataset collection, then applies preprocessing steps including mean imputation, one-hot encoding, Min-Max normalization, and SMOTE-based class balancing.

Three models—LR, RF, and XGBoost—are trained and optimized using the MPA. SHAP-based explainability is provided for global, local, and feature interaction analysis. Rigorous performance metrics ensure robust and interpretable diabetes prediction.

### 4.1. Comparative ML Model Development for Diabetes Prediction

The comparative model-building phase is a crucial component of the proposed XML framework. It compares several classification algorithms under the same experimental setup to identify the best, most stable, and interpretable model. The framework uses a mixture of interpretable and high-performance models by considering baseline, ensemble, and gradient boosting approaches. For fairness, all models are trained on the same preprocessed dataset and evaluated on unseen test data using uniform metrics.

#### 4.1.1. Baseline Model: Logistic Regression for Clinical Interpretability

**Figure 2:**
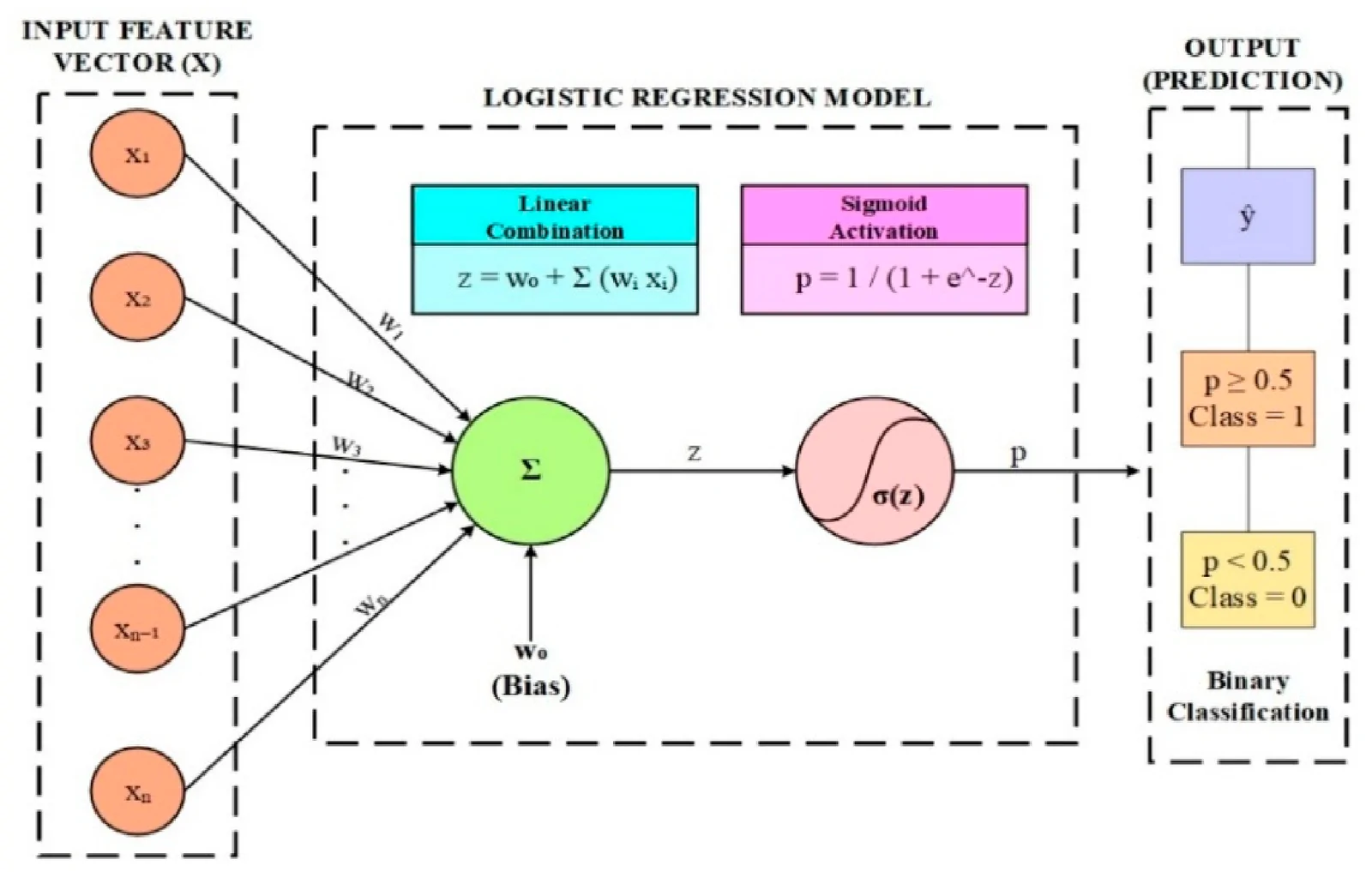
Architecture of LR for diabetes prediction.

LR serves as the baseline classifier in the proposed XML framework. It enables straightforward comparison with sophisticated ensemble and boosting approaches through a statistically sound and interpretable baseline. Clinical features are weighted and combined to produce a decision score, which is mapped to a probability via a sigmoid function to classify diabetic and non-diabetic patients (Figure 2).

LR estimates the probability of diabetes by applying a sigmoid function to a linear combination of clinical characteristics, as indicated by Equation (1):

(1) *P*(*y* = 1|*x*) = 1/(1 + *exp*(−(*β*0 + *β*1*x*1 + … + *βnxn*)))

where P(y = 1|x) is the probability of the diabetic condition, β0 is the intercept, β1, …, βn are the feature weights, x1, …, xn are the clinical features, and *n* is the number of features. The predicted probability is transformed into a binary class label as per Equation (2):

(2) *ŷ* = 1 *if*
*P^* ≥ 0.5, *else* 0

where ŷ is the predicted class label (1 = diabetic, 0 = non-diabetic) and P^ is the predicted probability. The feature influence is interpreted in terms of an odds ratio defined in Equation (3):

(3) *Odds Ratio* = *exp*(*βi*)

where βi is the coefficient of feature i. Positive coefficients correspond to increased risk of diabetes, and negative ones to protective effects.

#### 4.1.2. Ensemble Learning Model: Random Forest for Robust Prediction

**Figure 3:**
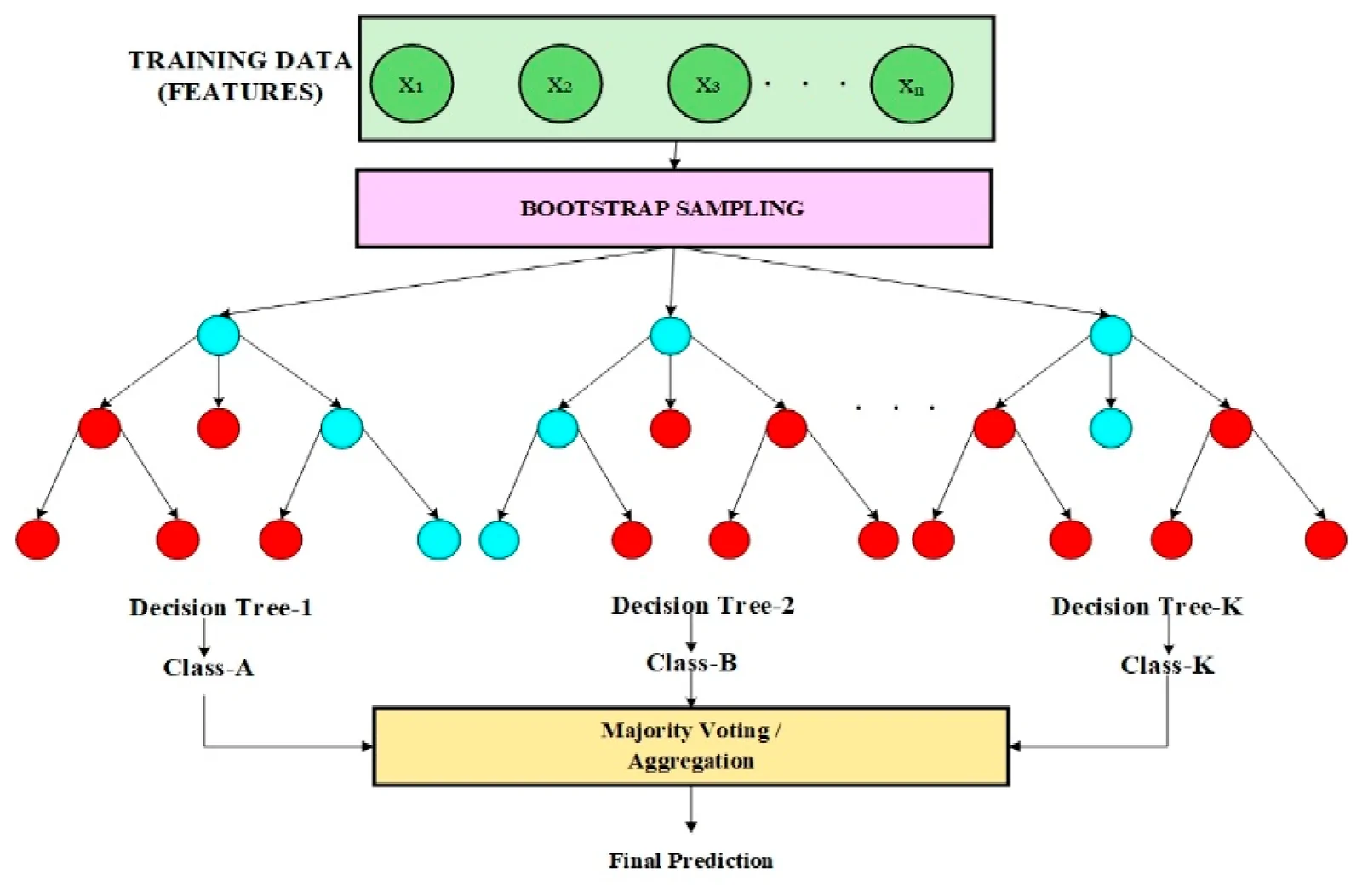
RF-based ensemble framework for robust classification.

RF is included to advance the stability and accuracy of the proposed diabetes prediction model, and to reduce overfitting by bagging multiple decision trees from diverse data subsets. It gains robustness for noisy and heterogeneous clinical data by consolidating various decision boundaries. In this work it serves as an intermediate model between LR and XGBoost. Figure 3 illustrates the RF structure in which multiple decision trees are trained using bootstrap-sampled subsets and final predictions are made by majority voting.

RF performs bootstrap sampling to create several training subsets with replacement, as in Equation (4):

(4) *Db* = {*x*1, *x*2, …, *xn*} *sampled with replacement from D*

where Db is the bootstrap dataset, *n* is the number of samples, and D is the initial dataset. The final prediction is determined by majority voting, as in Equation (5):

(5) *ŷ* = *mode*{*h*1(*x*), *h*2(*x*), …, *hT*(*x*)}

where ht(x) is the prediction from tree t over T total trees. In binary classification, RF node splitting employs the Gini Index to assess impurity, as in Equation (6):

(6) *Gini* = 1 − (*p*0^2^ + *p*1^2^)

where p0 and p1 represent the probabilities for non-diabetic and diabetic categories. Smaller Gini scores indicate purer subsets and better class separation.

#### 4.1.3. Gradient Boosting Model: XGBoost for High-Performance Classification

**Figure 4:**
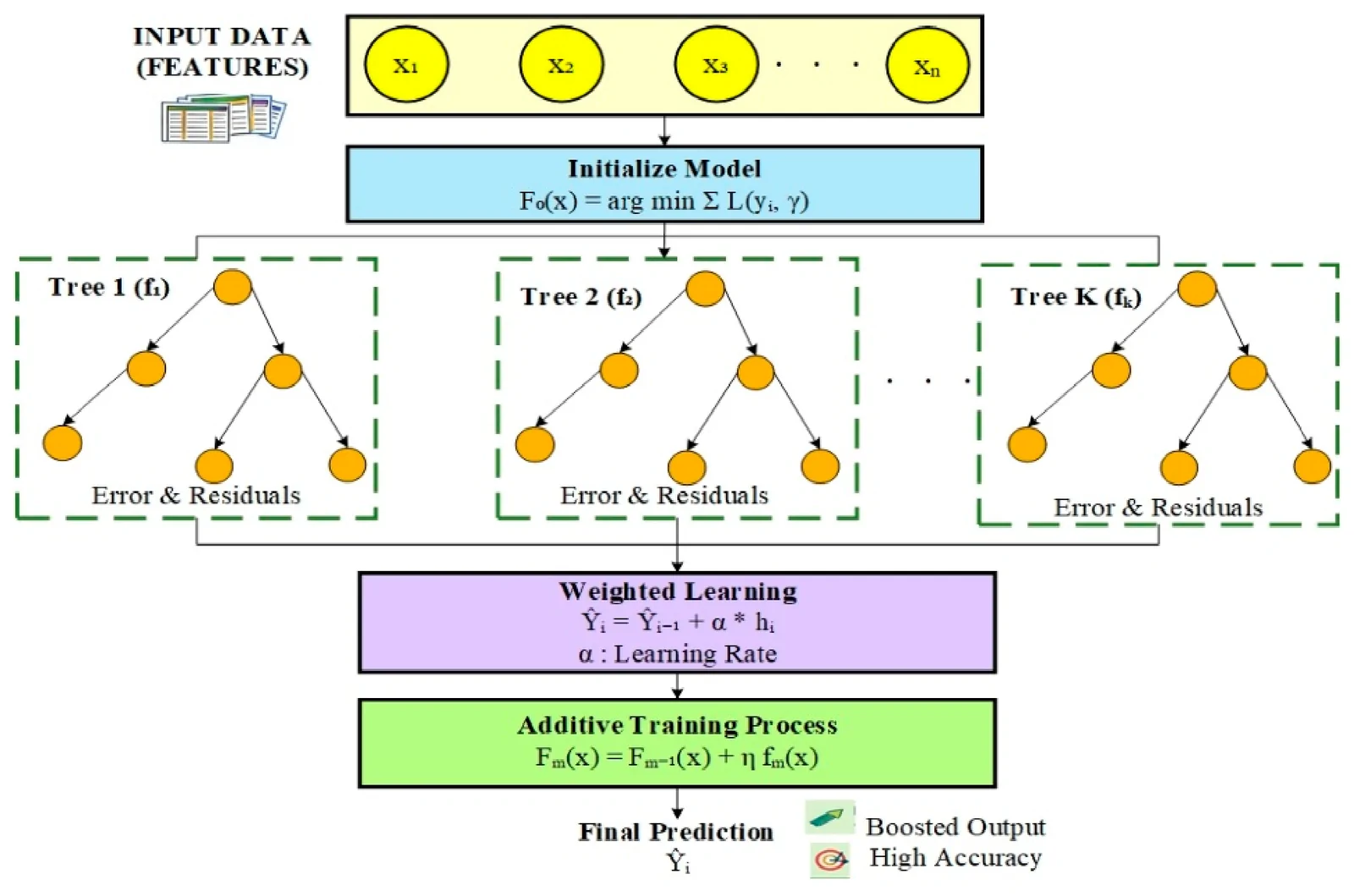
XGBoost architecture diagram for high-performance predictive modeling.

XGBoost operates as the high-performance gradient boosting classifier in the proposed framework. Its key purpose is to achieve the highest predictive accuracy by sequentially reducing classification errors via additive learning. Unlike bagging-based models, XGBoost trains algorithms (Algorithms 1–3) step by step and minimizes any differentiable loss function, making it suitable for structured clinical data with nonlinear relations. Figure 4 shows the framework of XGBoost in which decision trees are iteratively trained and added to the ensemble.

XGBoost makes its final prediction by combining weak learners, as in Equation (7):

(7) *ŷi* = ∑(*t* = 1 *to*
*T*) *ft*(*xi*), *ft* ∈ *K*

where T is the total boosting iterations, K is the space of regression trees, and ft is the prediction of the t-th tree. The regularized objective function is defined in Equation (8):

(8) *Obj* = ∑*i l*(*yi*, *ŷi*) + ∑*t Ω*(*ft*)

where l is the loss function, yi is the observed label, ŷi is the predicted output, and Ω is the regularization term for model complexity control, as in Equation (9):

(9) *Ω*(*f*) = *γT* + (1/2)*λ*∑*j wj*^2^

where γ is the leaf penalty, T is the number of leaves, wj is the leaf weight, and λ is the L2 regularization parameter.
**Algorithm** **1.** Comparative Machine Learning Model Development for Diabetes Prediction.Input: Pre-processed dataset D Output: Trained models, predictions, and best performing model Step 1: Load D; partition into D_train and D_test (stratified split). Step 2: Define LR (baseline), RF (ensemble), and XGBoost (gradient boosting). Step 3: Train LR on D_train using linear probabilistic relationships. Step 4: Train RF using bootstrap aggregation and majority voting. Step 5: Train XGBoost using sequential additive learning. Step 6: For each model, generate probability outputs for D_test;    convert to binary labels (0 = non-diabetic, 1 = diabetic). Step 7: Evaluate each model: Accuracy, Precision, Recall, F1, ROC-AUC. Step 8: Compare models; select best performer. Step 9: Return best model, predictions, and comparative performance results.

### 4.2. Hyperparameter Optimization Using Marine Predators Algorithm

The hyperparameter tuning step enhances the prediction accuracy and generalization of RF and XGBoost. Rather than manual tuning, MPA efficiently searches for optimal hyperparameters through a balanced exploration–exploitation strategy. LR is not optimized and instead serves as a fixed baseline for comparisons.

#### 4.2.1. Optimization Problem Definition

Hyperparameter tuning is formulated as an optimization problem, as indicated in Equation (10):

(10) *maximize F*(*θ*) = *w*1·*AUC* + *w*2·*F*1

where θ is the vector of hyperparameters, F(θ) is the fitness function, and w1, w2 are weight coefficients.

#### 4.2.2. MPA Mechanism

MPA is a population-based metaheuristic inspired by marine predator–prey interactions. It consists of three stages: high-velocity (exploration), unit-velocity (transition), and low-velocity (exploitation under Lévy/Brownian motion). The update rule is stated in Equation (11):

(11) *xi*’ = *xi* + *α* · (x* − *xi*) + *R*

where xi is the current position, xi’ is the new position, x* is the best solution, α is a stochastic factor, and R is a random disturbance term.

#### 4.2.3. Exploration–Exploitation Control Strategy

MPA employs a dynamic control factor, expressed in Equation (12):

(12) *CF* = (1 − *t*/*T*)^2^*^t^*^/^*^T^*

where CF is the control factor, t is the current iteration, and T is the maximum number of iterations.

#### 4.2.4. Fitness Evaluation Strategy

Every hyperparameter setting is tested using k-fold cross-validation, as in Equation (13):

(13) *F*(*θ*) = (1/*K*) ∑(*k* = 1 *to K*) *Sk*(*θ*)

where F(θ) is the aggregate performance, Sk denotes the assessment score of the k-th fold, and K is the total number of folds.
**Algorithm** **2.** MPA-Based Hyperparameter Optimization for Diabetes Prediction.Input: D_train, RF and XGBoost models Output: Optimized hyperparameters θ* for RF and XGB Step 1: Define hyperparameter search spaces for RF and XGBoost. Step 2: Initialize MPA: population size N, max iterations T. Step 3: Randomly initialize candidate solutions; assign to hyperparameter configs. Step 4: For each candidate, train model, perform K-fold CV, compute fitness. Step 5: Identify candidate with highest fitness as global best θ*. Step 6: For t = 1 to T:   6.1 Compute control factor CF.   6.2 Update candidate positions using MPA rule.   6.3 Enforce hyperparameter bounds.   6.4 Re-evaluate fitness; update global best if improved. Step 7: Apply optimized θ* to RF and XGB; generate final models.

### 4.3. Explainable Artificial Intelligence Using SHAP

An XAI stage is incorporated into the proposed diabetes prediction model to increase comprehension, openness, and credibility. Since ensemble approaches like RF and XGBoost operate as black-box models, SHAP is utilized to clarify predictions by representing the contribution of each clinical attribute toward the prediction of diabetes, guaranteeing both high accuracy and clinical interpretability.

#### 4.3.1. SHAP-Based Explainability Framework

SHAP is grounded in cooperative game theory, in which each feature is viewed as a participant influencing the outcome. The SHAP value formulation is provided in Equation (14):

(14) *φi* = ∑(*S*⊆*M*\{*i*}) [|*S*|!(|*M*| − |*S*| − 1)!/|*M*|!] [*fS*∪{*i*}(*x*) − *fS*(*x*)]

where M is the total number of attributes, S is a feature subset, fS is the model estimated using subset S, and φi is the SHAP value of feature i.

#### 4.3.2. Global Interpretability Analysis

Global feature importance is computed as the mean absolute SHAP value, as in Equation (15):

(15) *Ii* = (1/*n*) ∑(*j* = 1 *to n*) |*φi*(*xj*)|

where *n* is the number of observations, Ii is the global significance of feature i, and φi(xj) is the SHAP value for feature i on sample xj.

#### 4.3.3. Local Explanation Mechanism

SHAP explains predictions at the individual level, as in Equation (16):

(16) *f*(*x*) = *φ*0 + ∑(*i* = 1 *to M*) *φi*

where M is the number of features, φ0 is the base prediction, φi is the contribution of feature i, and f(x) is the final model prediction.

#### 4.3.4. Feature Interaction Analysis

SHAP also captures interaction effects between clinical variables such as the glucose–BMI relationship, where complex dependencies between features are revealed. SHAP-based explainability contributes to transparency and understanding of individual feature contributions, enabling clinically interpretable diabetes predictions for real-world healthcare use.

## 5. Experimental Setup

### 5.1. Dataset Acquisition

The diabetes outcome variable was obtained directly from the publicly available Kaggle dataset as a predefined binary label. However, the original label-generation mechanism is fully documented by the dataset source; therefore, it is unable to be independently verified whether HbA1c level, blood glucose level, or other clinical variables contributed directly or indirectly to the construction of the target label. Accordingly, this study treats the provided outcome as recorded diabetes status and formulates the task as supervised classification rather than prospective early diabetes risk prediction.

This consequently contributes substantially to classification performance. Therefore, the proposed framework is intended as a supervised diabetes classification model based on routinely available clinical and demographic attributes rather than a model for predicting future diabetes risk before laboratory assessment. All input variables, including HbA1c level and blood glucose level, were treated as observational features available at the time of patient evaluation. SMOTE was applied only to the training data during model development to prevent data leakage.

The proposed work exploits the publicly accessible Diabetes Prediction Dataset [42] for binary classification. It contains nearly 100,000 samples, consisting of demographic, clinical, and lifestyle features significant for assessing the risk of diabetes. The dataset is class-imbalanced, as shown in Table 1. SMOTE is applied only on the training data to generate synthetic diabetic samples and balance classes without data leakage. The dataset is standardized to 20,000 records after preprocessing and SMOTE augmentation for computational tractability and fair comparison.

**Figure 5:**
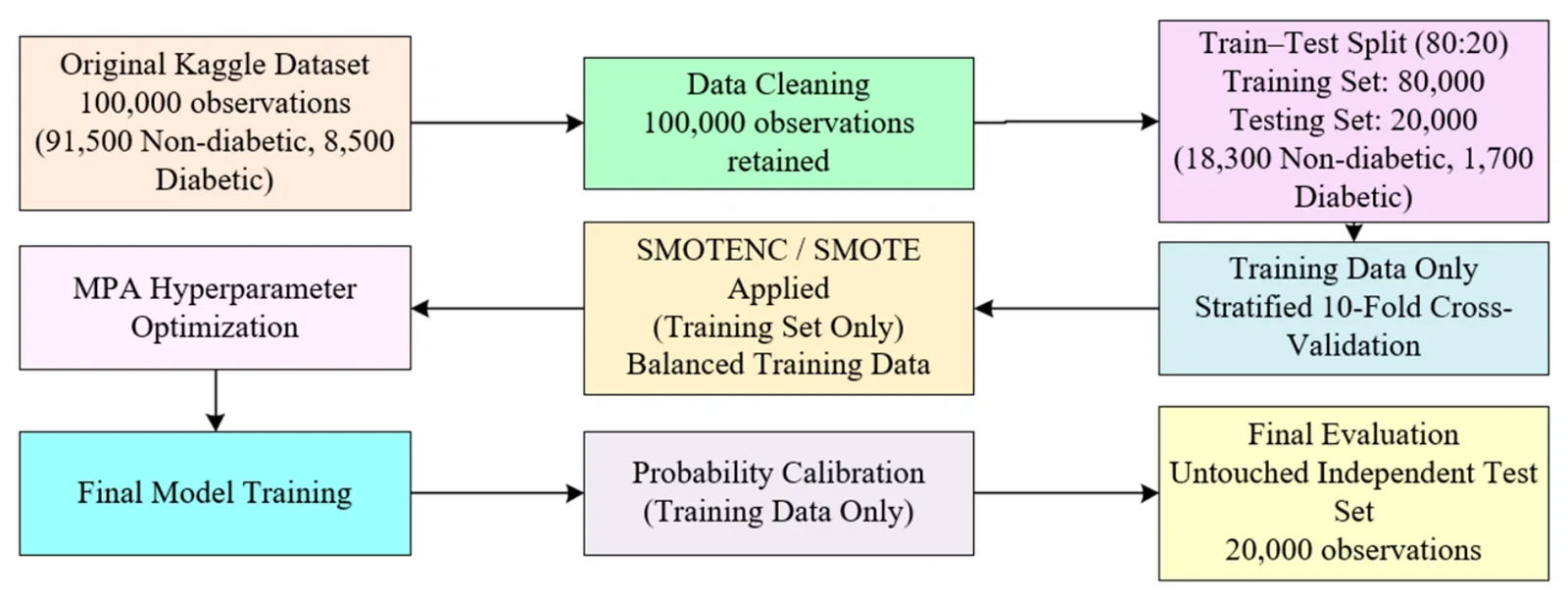
Sample Allocation for Training, Validation, and Testing.

Figure 5 illustrates the complete experimental pipeline adopted in this study. Following duplicate removal, the dataset was partitioned into training and untouched independent test sets. Isolation Forest-based outlier removal, hyperparameter optimization, stratified 10-fold cross-validation with SMOTE, final model training, and probability calibration were performed exclusively on the training data. The untouched independent test set was preserved throughout model development and used only once for the final evaluation of classification, calibration, statistical significance, and error analysis.

Table 1 summarizes the complete sample flow adopted in the proposed framework. After duplicate removal, the dataset was partitioned into training and untouched independent test sets. Isolation Forest outlier removal, hyperparameter optimization, cross-validation, SMOTE, and probability calibration were performed exclusively on the training data, whereas the independent test set remained unchanged and was used only once for the final evaluation of classification, calibration, and statistical performance.

Table 2 summarizes the class distribution throughout the experimental pipeline. SMOTE was applied exclusively to the training set after the initial stratified 80:20 split, whereas the independent test set remained unchanged, retained its original class distribution, and contained no synthetic samples.

The study utilized the publicly available Diabetes Prediction Dataset obtained from Kaggle, which contains approximately 100,000 anonymized patient records represented by demographic, clinical, lifestyle, anthropometric, and laboratory variables. The available demographic variables include age and gender, while the clinical history comprises hypertension and heart disease status. Lifestyle information is represented by smoking history, and anthropometric assessment is captured through body mass index (BMI). Laboratory measurements include HbA1c level and blood glucose level, both of which are routinely used in diabetes assessment. The target variable is a predefined binary diabetes status label provided by the dataset source, where each record is categorized as either diabetic or non-diabetic. The original Kaggle documentation provides limited information regarding participant recruitment, inclusion criteria, measurement protocols, and the underlying data-generation process; therefore, the dataset should be regarded as a public benchmark dataset for methodological evaluation rather than a clinically validated cohort representative of routine healthcare populations. Prior to model development, the dataset underwent data quality assessment to identify incomplete or inconsistent records. Records containing missing or invalid values were excluded during preprocessing to ensure a complete dataset for model training and evaluation. Following preprocessing, 20,000 samples were selected for experimentation, maintaining class balance through stratified sampling before the application of SMOTE exclusively to the training data. Accordingly, the reported findings should be interpreted as demonstrating methodological performance on this benchmark dataset and should not be considered evidence of real-world clinical validity.

A stratified random sample of 20,000 records was selected from the original ~100,000 samples to ensure computational feasibility during repeated stratified 10-fold cross-validation and MPA-based hyperparameter optimization. This sampling preserved the original class distribution and statistical characteristics of the dataset, thereby maintaining representativeness while enabling fair and efficient model evaluation. SMOTE was then applied exclusively to the training data to handle class imbalance without introducing data leakage. The proposed framework was developed and evaluated using a single publicly available Kaggle diabetes dataset, which served as a standardized benchmark for model development and comparison. Model performance was assessed through stratified 10-fold cross-validation to provide a reliable estimate of internal validation performance. However, no independent external clinical dataset was available for validation. Consequently, the reported results should be interpreted as evidence of methodological effectiveness rather than confirmed generalizability across diverse clinical populations. Future work will focus on validating the proposed framework using independent multi-center datasets to further assess its robustness and clinical applicability.

### 5.2. Data Preprocessing

Healthcare data quality heavily influences the reliability of prediction systems. Missing values, heterogeneous records, and imbalanced classes in healthcare datasets lead to unreliable predictions. Hence, preprocessing increases data quality, feature uniformity, and framework effectiveness by standardizing attributes, balancing classes, encoding categorical variables, and handling missing data.

#### 5.2.1. Mean Imputation for Missing Value Handling

Mean imputation substitutes the mean value for any absent attribute, as in Equation (17):

(17) *μ* = (1/*n*) ∑(*i* = 1 *to n*) *xi*

where μ is the mean, *n* is the total number of samples, and xi is the i-th sample value.

#### 5.2.2. One-Hot Encoding for Categorical Feature Transformation

Categorical features are converted to binary numerical vectors, as shown in Equation (18):

(18) *e*(*x*) = [1 *if x* = *ck else* 0] *for k* = 1,…,*K*

where e(x) is the encoded representation of categorical variable x.

#### 5.2.3. Min-Max Normalization for Feature Scaling

Min-Max normalization transforms numeric features into a fixed range, as in Equation (19):

(19) *x*’ = (*x* − *xmin*)/(*xmax* − *xmin*)

where x’ is the normalized value of x, and xmin and xmax are the feature’s minimum and maximum values, respectively. The preprocessing order was designed to prevent information leakage and ensure valid sample generation. The dataset was first partitioned into training and independent test sets. All preprocessing operations, including categorical feature handling, SMOTENC-based oversampling, hyperparameter optimization, and probability calibration, were performed exclusively on the training data. The independent test set retained its original class distribution and was used only once for the final evaluation. To preserve the integrity of categorical attributes during class balancing, the Synthetic Minority Over-sampling Technique for Nominal and Continuous features (SMOTENC) was employed instead of conventional SMOTE. Categorical feature indices were specified before oversampling, enabling SMOTENC to generate synthetic minority samples while maintaining valid categorical values and avoiding fractional or implausible category combinations. This approach is more appropriate for datasets containing both continuous and categorical variables.

Table 3 summarizes the preprocessing sequence adopted in this study. Categorical variables were handled using SMOTENC, and all preprocessing and model development procedures were performed exclusively on the training data. The untouched independent test set remained unchanged until the final evaluation.

#### 5.2.4. SMOTE for Class Imbalance Handling

SMOTE creates artificial minority class samples to balance the class distribution, as in Equation (20):

(20) *xnew* = *xi* + *λ* · (*xnn* − *xi*), *λ* ∈ [0, 1]

where xnew is the new sample, xi denotes a minority instance, xnn is its nearest neighbor, and λ is the interpolation factor (Algorithm 3).
**Algorithm** **3.** SMOTE for Class Imbalance Handling.Input: X_min (minority class), N (synthetic samples required), k (nearest neighbors) Output: X_syn (synthetic minority class samples) Step 1: Initialize X_syn = ∅. Step 2: For each xi ∈ X_min:   2.1 Locate k nearest neighbors in X_min. Step 3: For i = 1 to N:   3.1 Randomly select xi from X_min.   3.2 Randomly select xnn from k nearest neighbors of xi.   3.3 Generate: xnew = xi + λ × (xnn − xi), λ ∈ [0, 1].   3.4 Add xnew to X_syn. Step 4: Return X_syn.

### 5.3. Data Splitting Strategy

#### 5.3.1. Training and Testing Split

The dataset is partitioned into separate training and testing sets to prevent data leakage and facilitate trustworthy evaluation, as formulated in Equation (21):

(21) *D_train* = 0.80 × *D*, *D_test* = 0.20 × *D*

where D_train is the training portion, D_test is the testing portion, and D is the complete dataset. An initial stratified 80:20 train–test split was performed to separate the training and independent testing datasets. The independent test set was isolated before any preprocessing or model development and was not used during feature preprocessing, hyperparameter optimization, or cross-validation. Stratified 10-fold cross-validation was then performed exclusively on the training set. Within each fold, preprocessing and SMOTE were fitted only on the corresponding training partition before model fitting. After hyperparameter optimization, the final model was retrained using the complete training dataset and evaluated once on the untouched independent test set.

Algorithm 4 presents the experimental workflow adopted in this study. The dataset is first partitioned into independent training and testing sets using a stratified 80:20 split. All preprocessing operations, including mean imputation, one-hot encoding, Min-Max normalization, and SMOTE, are performed exclusively within the training folds during stratified 10-fold cross-validation to prevent data leakage. The optimal hyperparameters are selected based on the mean cross-validation ROC-AUC, after which the final model is retrained using the complete training dataset and evaluated once on the untouched independent test set. This procedure ensures fair model evaluation and reliable performance estimation.
**Algorithm 4.** The Pipeline for Data Splitting, Cross-Validation, and SMOTE-Based Model DevelopmentInput: Complete dataset D Step 1: Perform stratified 80:20 train-test split  → Training set (80%)  → Independent Test set (20%) Step 2: Keep the independent test set completely untouched. Step 3: For each fold of stratified 10-fold cross-validation:   (a) Fit mean imputation using training fold only.   (b) Apply imputation to validation fold.   (c) Fit one-hot encoding using training fold only.   (d) Apply encoding to validation fold.   (e) Fit Min-Max normalization using training fold only.   (f) Apply normalization to validation fold.   (g) Apply SMOTE only to the training fold.   (h) Train classifier.   (i) Validate on untouched validation fold. Step 4: Select optimal hyperparameters using mean ROC-AUC. Step 5: Retrain model using complete training data. Step 6: Evaluate once on the untouched independent test set. Output: Cross-validation performance Independent test-set performance

#### 5.3.2. Stratified 10-Fold Cross-Validation

Stratified 10-fold cross-validation is applied to increase robustness and reduce overfitting while maintaining class distribution across folds, as in Equation (22):

(22) *V* = (1/*K*) ∑(*k* = 1 *to K*) *Sk*, *K* = 10

where V is the validation score and Sk is the score of fold k. This technique ensures better stability, generalization, and fairer comparative assessment.

### 5.4. Experimental Environment

Table 4 lists the hardware and software environment used in this study.

### 5.5. Hyperparameter Configuration

Hyperparameter optimization and performance estimation were conducted using separate data partitions to prevent optimistic performance estimates. The training set was first subjected to stratified 10-fold nested cross-validation, where the inner folds were used exclusively for MPA-based hyperparameter optimization and the outer folds were reserved for performance estimation. After selecting the optimal hyperparameters, the final model was retrained using the complete training dataset. The untouched independent test set was not involved in hyperparameter optimization or cross-validation and was evaluated only once after model selection.

Table 5 summarizes the validation strategy adopted in this study by clearly separating hyperparameter optimization from performance estimation. Hyperparameter selection was performed exclusively on the training data, while the final model was evaluated only once using the untouched independent test set to ensure an unbiased assessment of predictive performance.

In Table 6 Marine Predators Algorithm (MPA) optimization experiments were performed using a fixed random seed of 42. Candidate hyperparameter solutions were initialized using uniform random initialization within predefined search ranges and evaluated using identical stratified 10-fold cross-validation partitions throughout the optimization process. The optimization objective was to maximize the mean cross-validation ROC-AUC, with the F1-score used as a secondary criterion when equivalent ROC-AUC values were obtained. The optimization was performed with a population size of 10 and a maximum of 15 iterations, and the best-performing hyperparameter configuration was selected for final model training and evaluation.

Table 7 presents the lower and upper bounds of the XGBoost hyperparameters explored by the Marine Predators Algorithm during optimization. Candidate solutions were initialized within these predefined ranges, and the optimal hyperparameter combination was selected based on the highest mean ROC-AUC obtained through stratified 10-fold cross-validation.

**Figure 6:**
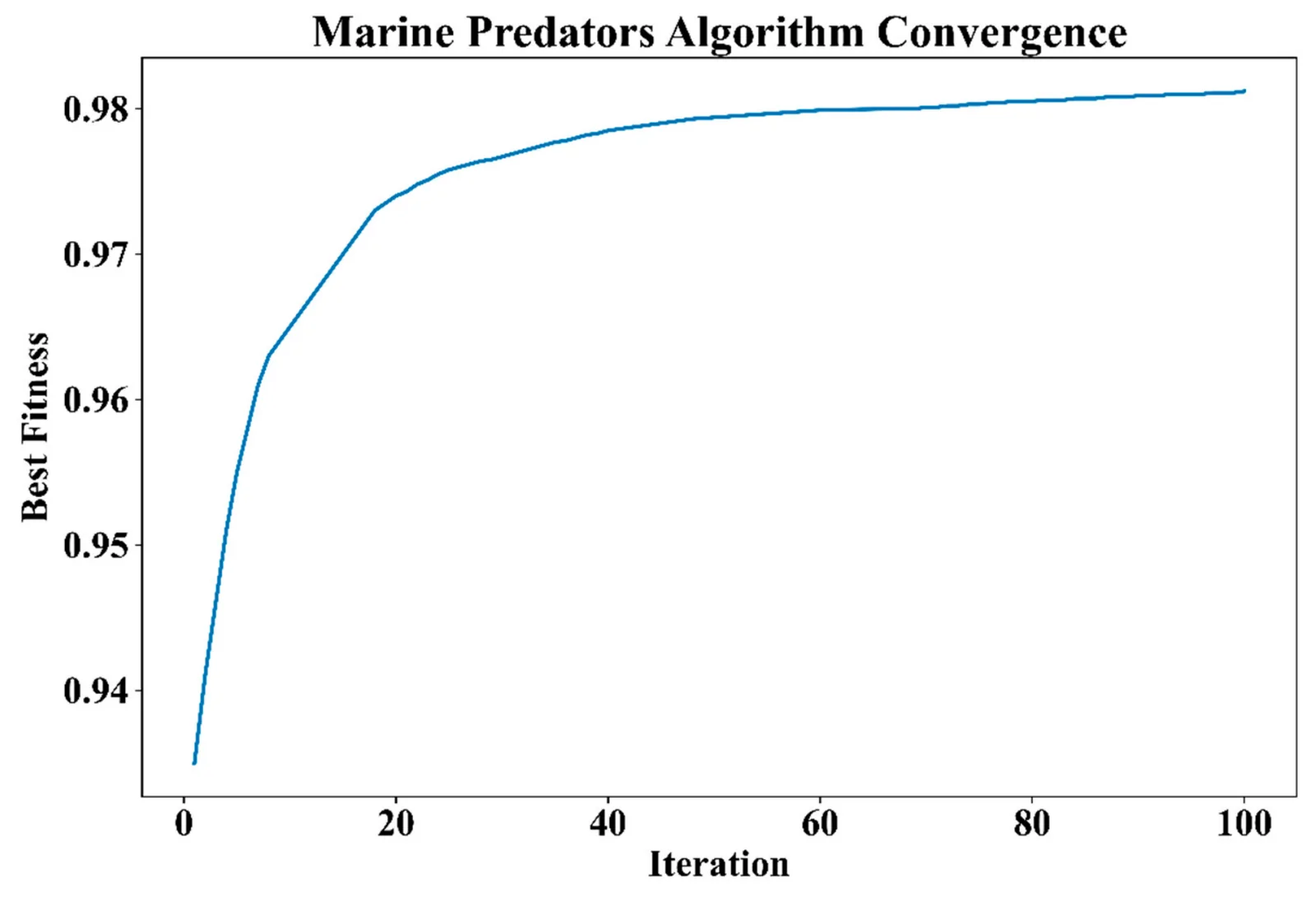
Convergence behavior of the Marine Predators Algorithm.

Figure 6 illustrates the convergence behavior of the Marine Predators Algorithm during the optimization of XGBoost hyperparameters. The fitness value improves rapidly during the initial iterations as the algorithm efficiently explores the search space and identifies promising hyperparameter combinations. After approximately the middle of the optimization process, the convergence curve stabilizes with only marginal improvements in subsequent iterations, indicating that the algorithm has converged to a stable near-optimal solution. This convergence behavior demonstrates that the selected optimization configuration effectively balances exploration and exploitation while maintaining computational efficiency for the proposed diabetes prediction framework.

Table 8 presents the optimal hyperparameter values identified by the Marine Predators Algorithm for the Random Forest and XGBoost classifiers. These parameter values were selected from the predefined search space by maximizing the mean ROC-AUC during stratified 10-fold cross-validation and were subsequently used for the final model training and independent test-set evaluation.

### 5.6. Performance Metrics

To estimate classification performance and robustness against imbalanced data, the framework employs the following metrics. Accuracy measures the proportion of correctly classified samples, as shown in Equation (23). Precision measures correctly predicted diabetic individuals among positive predictions, as shown in Equation (24). Recall (Sensitivity) measures how well the model detects true positive diabetics, as shown in Equation (25). F1-Score is the harmonic mean of Precision and Recall, as shown in Equation (26).

(23) *Accuracy* = (*TP* + *TN*)/(*TP* + *TN* + *FP* + *FN*)

(24) *Precision* = *TP*/(*TP* + *FP*)

(25) *Recall* = *TP*/(*TP* + *FN*)

(26) *F*1 = 2 × (*Precision* × *Recall*)/(*Precision* + *Recall*)

where TP, TN, FP, and FN are true positives, true negatives, false positives, and false negatives, respectively. The performance metrics reported in this study are categorized according to their evaluation protocol. The mean ROC-AUC obtained from stratified 10-fold cross-validation is reported separately from the ROC-AUC computed on the independent hold-out test set. Cross-validation results are presented as the mean ± standard deviation (SD), whereas the independent test-set ROC-AUC is obtained from the ROC curve generated using the final optimized model. Threshold-dependent metrics (accuracy, precision, recall, F1-score, and specificity) are computed after applying the default classification threshold of 0.5. The stratified 10-fold cross-validation yielded a mean ROC-AUC of 0.9948 ± SD for the proposed XGBoost model. In contrast, the independent hold-out test set produced an Accuracy of 0.9672, Precision of 0.9770, Recall of 0.9570, F1-score of 0.9671, and ROC-AUC of 0.9956. These values correspond to different evaluation protocols.

The objective function used by the MPA optimization algorithm was defined as a weighted combination of ROC-AUC and F1-score to balance discrimination ability and minority-class prediction performance. ROC-AUC shows the classifier’s ability to distinguish between classes across all thresholds, as shown in Equation (27). The Brier score measures calibration quality. The fitness function is expressed as:

(27) Fitness=0.7×(1−ROC−AUC)+0.3×(1−F1)

where ROC-AUC was assigned the primary weight (0.7) and F1-score was assigned a secondary weight (0.3). The optimization process minimized the fitness value, corresponding to maximization of both ROC-AUC and F1-score. In cases of similar fitness values, ROC-AUC was considered the primary selection criterion.

Table 9 summarizes the RandomizedSearchCV optimization settings, hyperparameter search spaces, and selected optimal configurations for Random Forest and XGBoost models. The optimization was performed using stratified 5-fold cross-validation with ROC-AUC as the scoring criterion and a fixed random seed of 42 to ensure reproducibility.

## 6. Results and Discussion

The proposed explainable diabetes prediction framework, incorporating LR, RF, and MPA-enhanced XGBoost, is analyzed in terms of performance, calibration, SHAP interpretation, ablation study, and statistical validation.

### 6.1. Dataset Distribution and Feature Analysis

**Figure 7:**
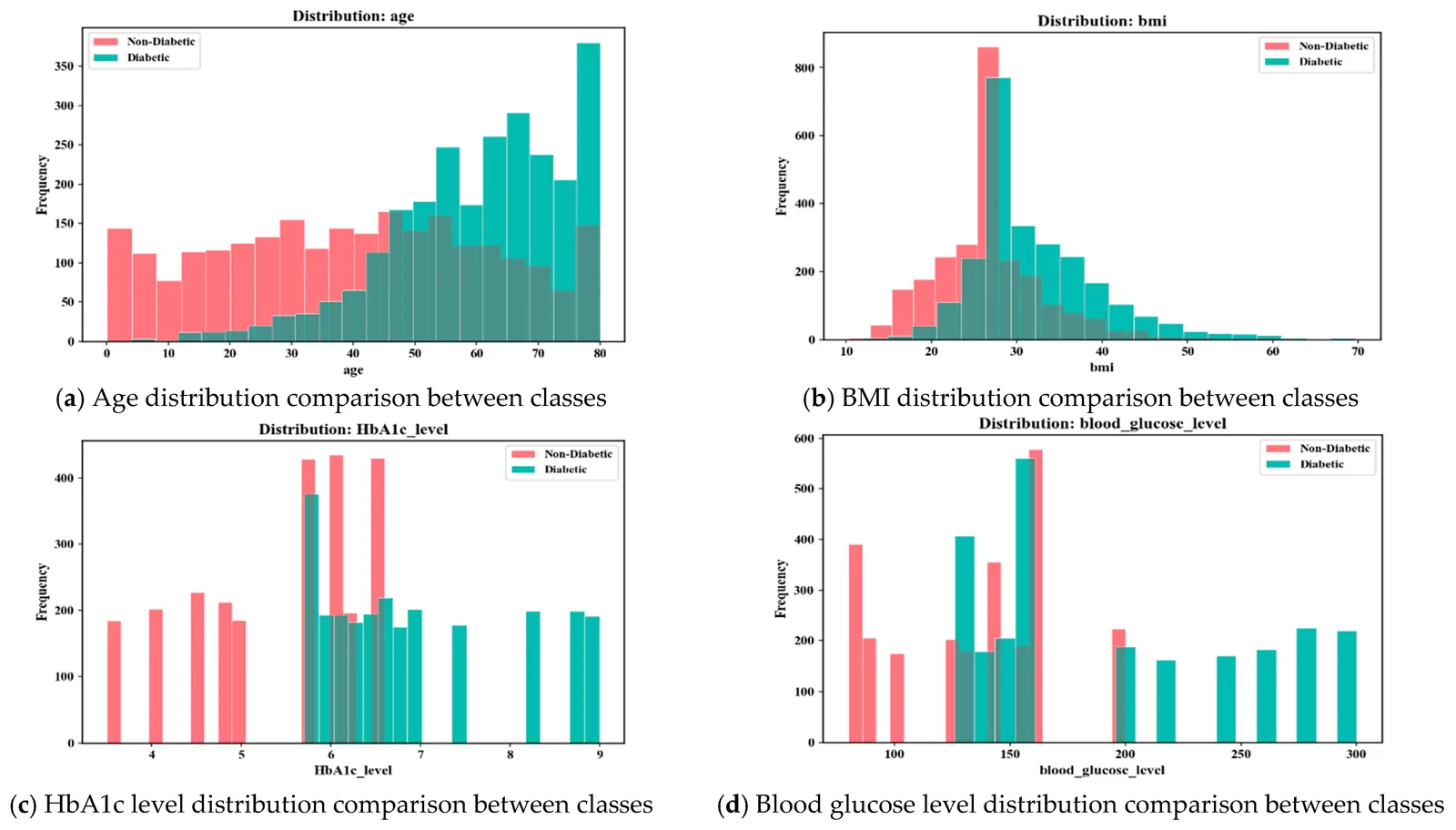
Feature distribution analysis: (**a**) age distribution comparison between classes; (**b**) BMI distribution comparison between classes; (**c**) HbA1c level distribution comparison between classes; (**d**) blood glucose level distribution comparison between classes.

Figure 7 shows the distribution of key clinical characteristics and the comparison between diabetic and non-diabetic classes. Diabetic patients predominate in the age group 50–80 years, and a BMI peak of 25–35 kg/m^2^ is observed for diabetics. HbA1c values for diabetic individuals are mostly in the range 6–9%, and blood glucose levels are mainly concentrated between 150–300 mg/dL. These discriminative patterns justify the use of these features in predictive models.

**Figure 8:**
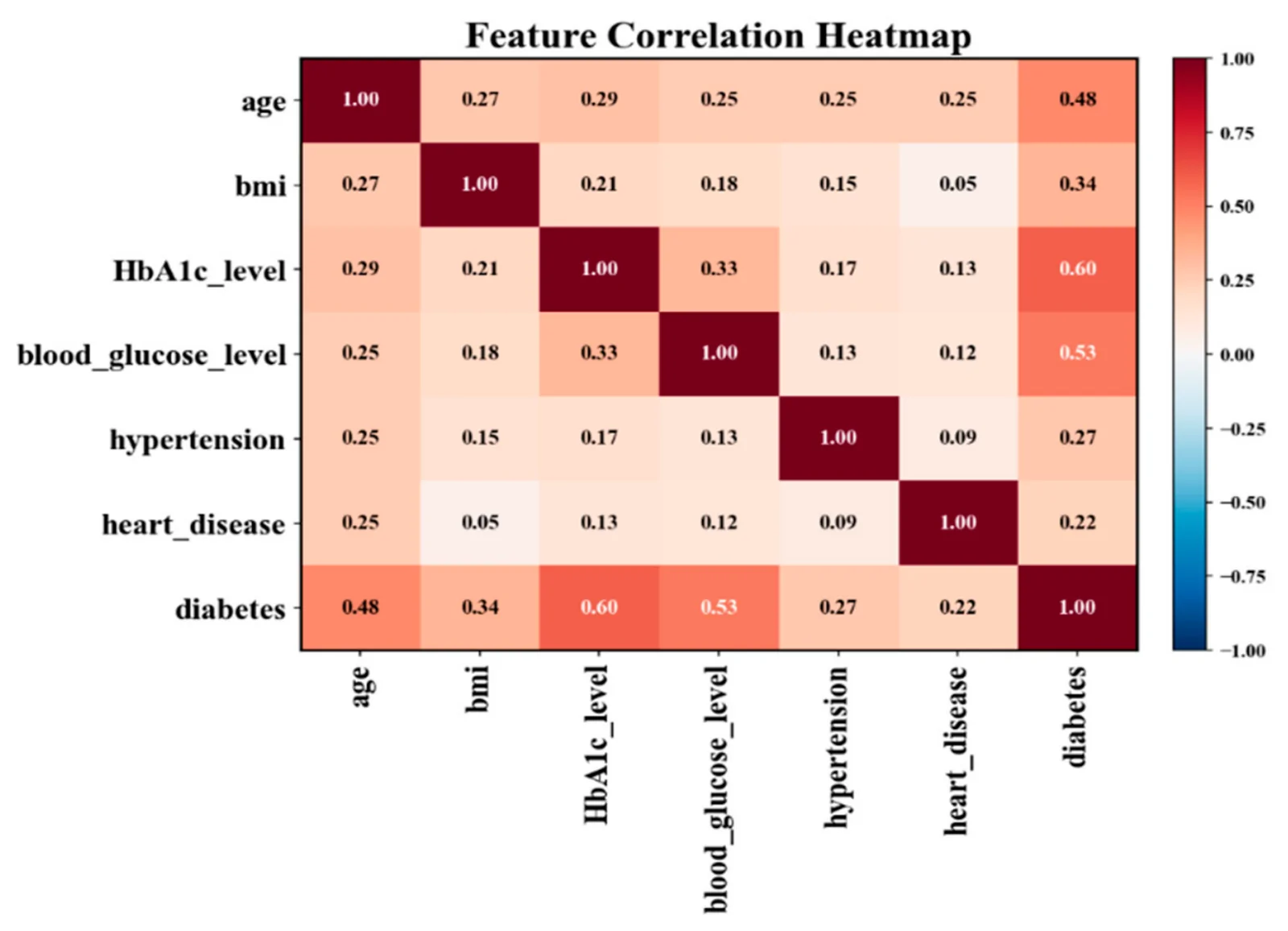
Correlation heatmap of clinical features.

Figure 8 shows a correlation matrix heatmap of the clinical features. Diabetes shows moderate positive associations with HbA1c_level (0.60), blood_glucose_level (0.53), age (0.48), and BMI (0.34), and weaker positive associations with hypertension (0.27) and heart_disease (0.22). HbA1c_level is also associated with blood_glucose_level (0.33). This analysis informs feature selection and model design.

### 6.2. Cross-Validation and Comparative Performance

**Figure 9:**
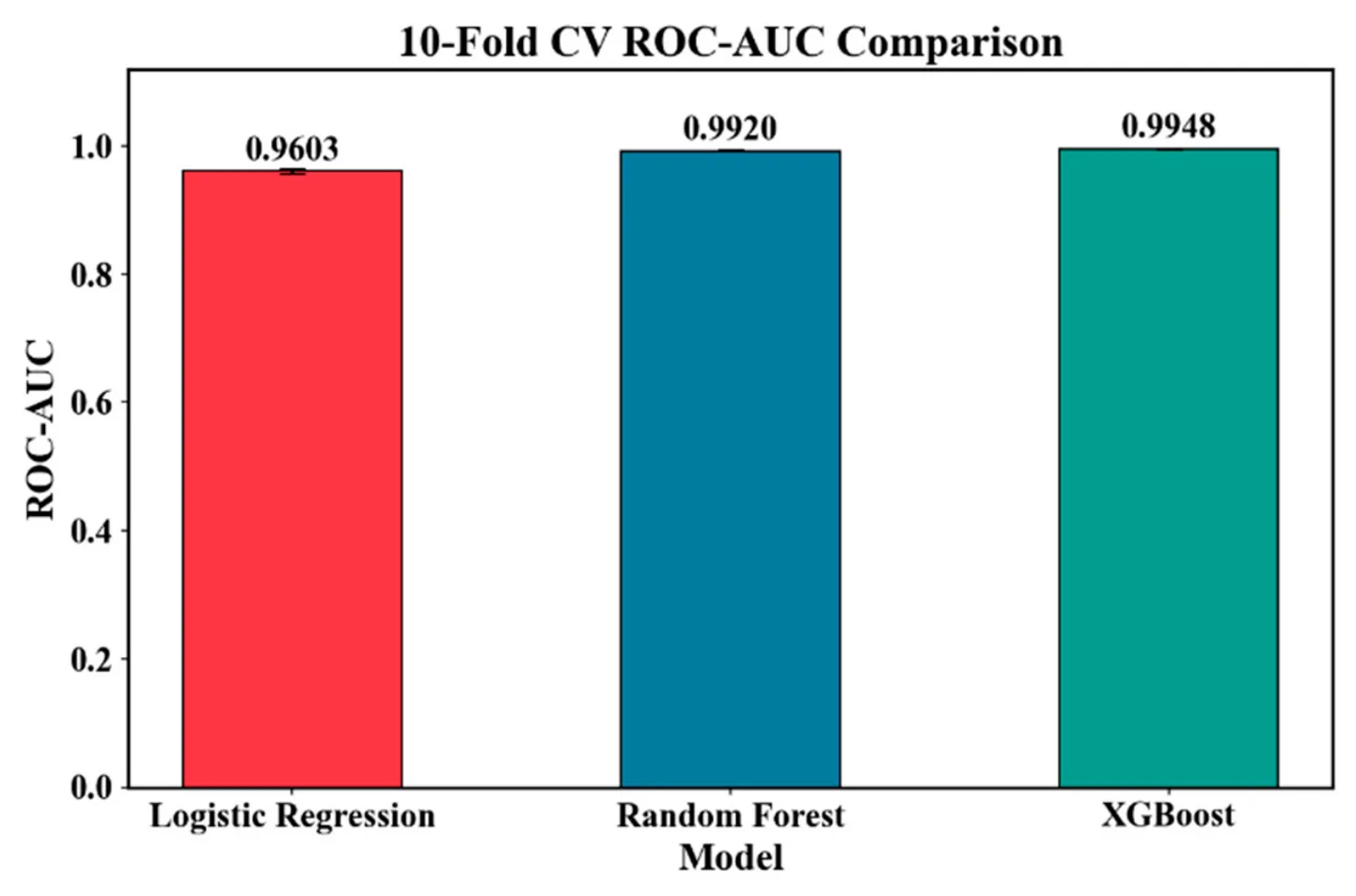
ROC-AUC performance of models via 10-fold cross-validation.

Figure 9 presents the stratified 10-fold cross-validation results. The proposed XGBoost model achieved a mean ROC-AUC of 0.9948 ± SD across the ten folds, outperforming RF (0.9920 ± SD) and LR (0.9603 ± SD). These values represent internal validation performance and are reported separately from the independent test-set evaluation.

The cross-validation results reported in this section represent the performance estimation stage and were obtained independently from the hyperparameter optimization process. Stratified cross-validation was performed only on the training data to evaluate model stability and generalization capability. The independent test set was not involved in cross-validation or parameter selection and was reserved exclusively for the final evaluation after model development.

Table 10 presents the stratified 10-fold cross-validation performance of the evaluated machine learning models. The reported values represent the fold-level mean, standard deviation, and 95% confidence interval for each evaluation metric, providing a robust estimate of model performance variability independently of the untouched test-set evaluation.

**Figure 10:**
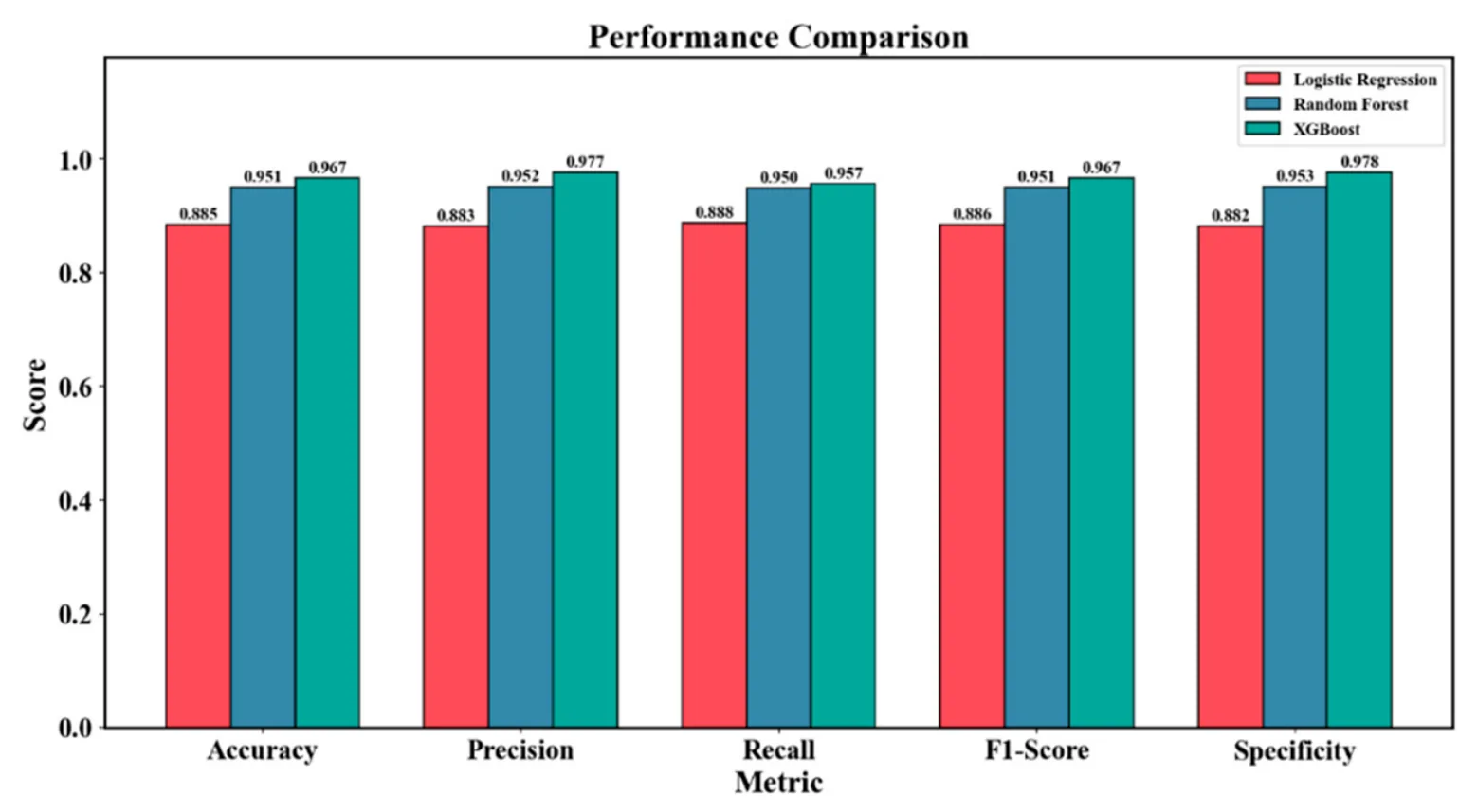
Comparative performance metrics of diabetes prediction models.

The performance values presented in Figure 10 correspond exclusively to the independent hold-out test set. Accordingly, the reported ROC-AUC of 0.9956 for the optimized XGBoost model differs from the cross-validation ROC-AUC (0.9948 ± SD) because the two values originate from different evaluation protocols.

**Figure 11:**
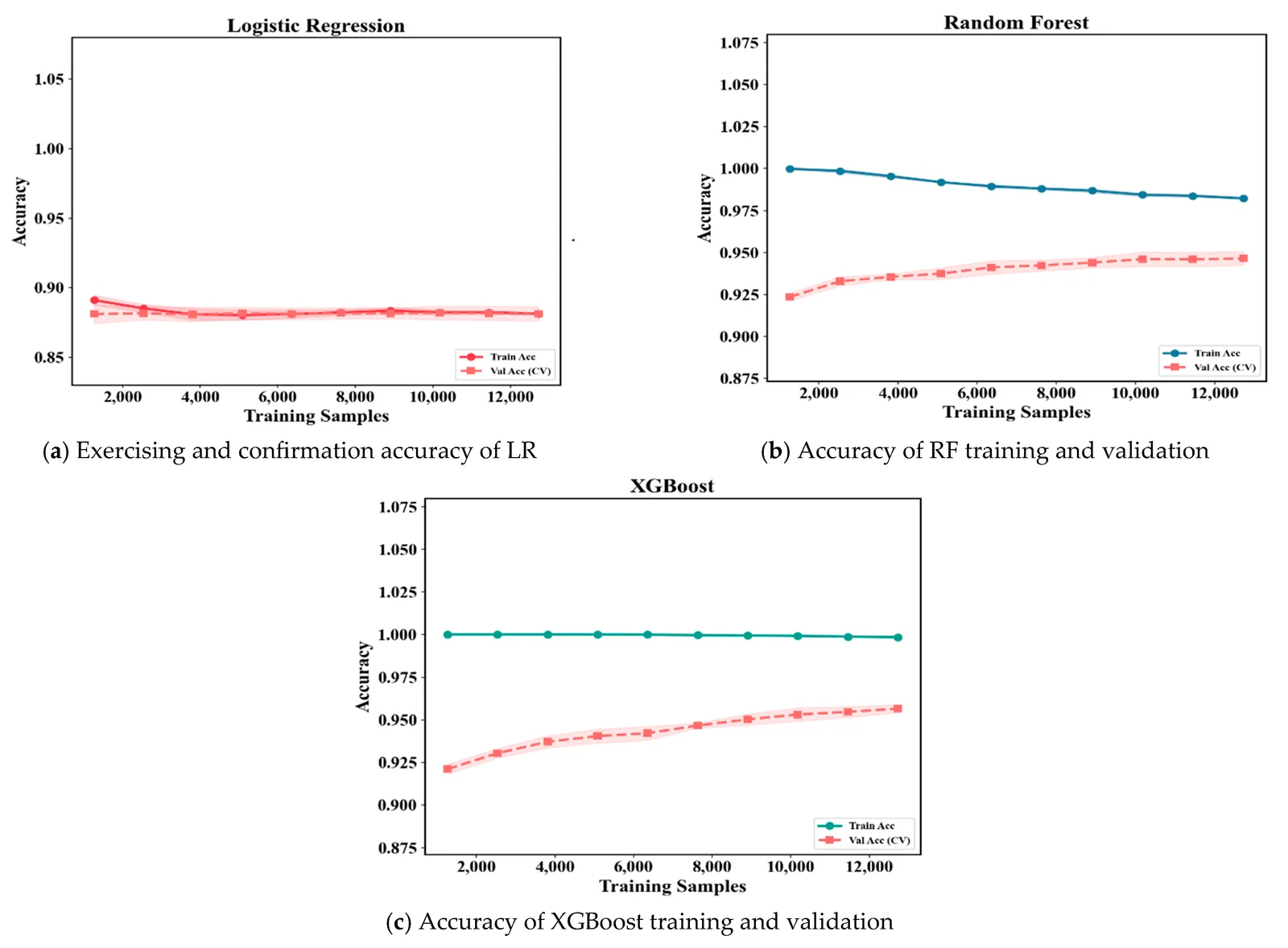
Learning curves: (**a**) LR; (**b**) RF; (**c**) XGBoost.

Figure 11 Shows the learning curves with training sample sizes varying from 2000 to 12,000. LR performs consistently with training and validation accuracy of ~0.890 and ~0.880. RF generalizes better with training accuracy ~0.998 and validation accuracy rising from 0.924 to 0.944. XGBoost shows the best stability, with validation accuracy increasing from 0.922 to 0.956.

### 6.3. Evaluation of Predictive Probability Calibration

Model calibration was evaluated on the independent hold-out test set without applying SMOTE to preserve the original class distribution. In addition to the Brier score, calibration-in-the-large (CITL), calibration slope, expected calibration error (ECE), and calibration curves were used to assess the agreement between predicted probabilities and observed outcomes. Since SMOTE was applied only during model training, probability calibration was reassessed after model optimization using post hoc probability calibration on the validation set to minimize calibration bias.

**Figure 12:**
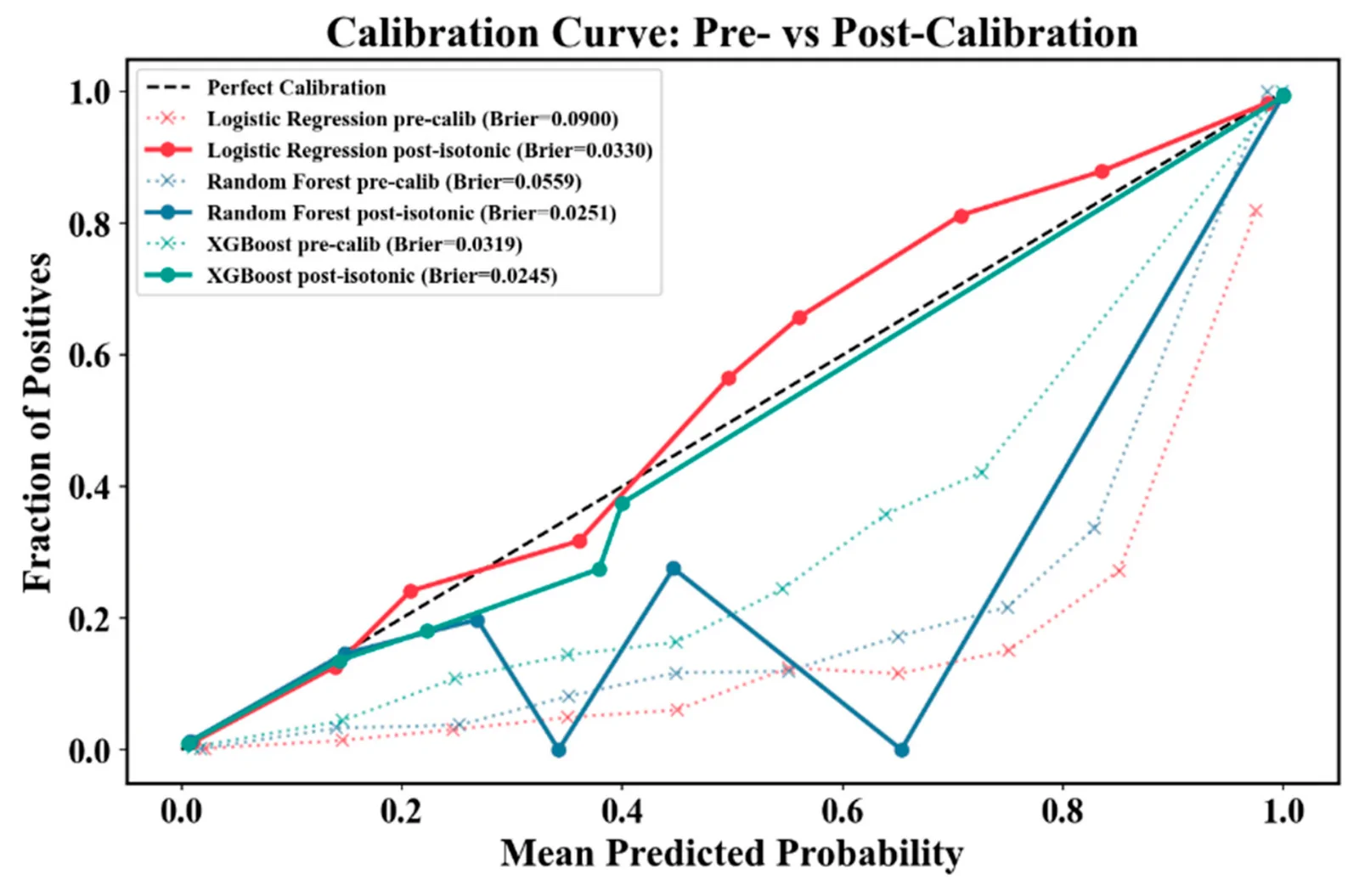
Calibration Curve of the Proposed MPA-XGBoost Model on the Untouched Independent Test Set (20,000 Observations).

Figure 12 illustrates the calibration performance of the evaluated machine learning models using the untouched independent test set containing 20,000 observations (18,300 non-diabetic and 1700 diabetic cases). The calibration curves compare the predicted probabilities with the observed outcome frequencies, where the diagonal reference line represents perfect calibration. Models with curves closer to the reference line demonstrate better agreement between predicted and observed probabilities. The proposed MPA-XGBoost model exhibits the closest alignment with the ideal calibration line, indicating improved probability estimation compared with the baseline models. These calibration results were obtained exclusively from the untouched independent test set after completion of model training and hyperparameter optimization, ensuring an unbiased assessment of probability reliability. Probability calibration was performed to improve the agreement between predicted probabilities and the observed outcomes in the benchmark dataset. The calibrated probabilities provide a more reliable representation of model confidence within the evaluated data.

Table 11 summarizes the calibration and classification performance of the evaluated machine learning models before and after post hoc calibration using the untouched independent test set. The post-calibration results demonstrate improved probability estimation, as reflected by reduced Brier scores and log loss, while maintaining comparable classification performance. Among the evaluated models, the proposed XGBoost model achieved the balance between predictive accuracy, discrimination, and calibration performance.

### 6.4. ROC and Precision–Recall Analysis

**Figure 13:**
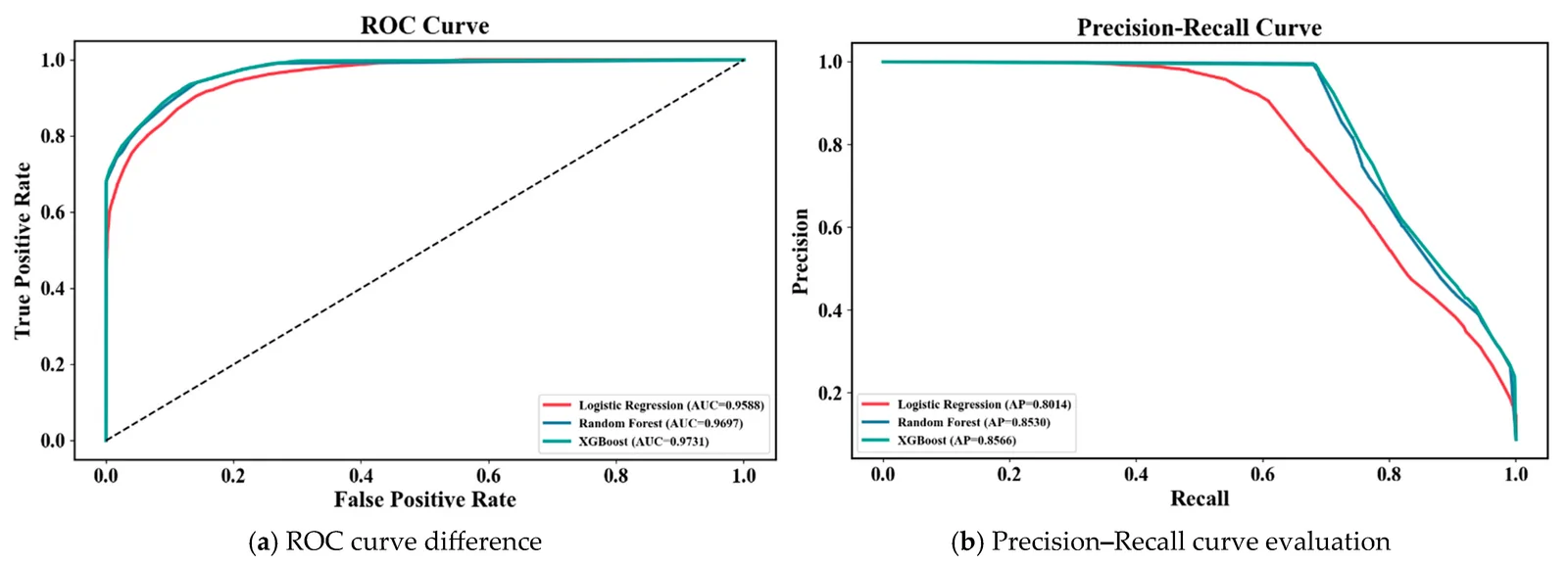
Comparison of classic performance analysis: (**a**) ROC curves; (**b**) Precision–Recall curves.

Figure 13 presents the ROC curves of the evaluated models using the untouched independent test set comprising 20,000 observations (18,300 non-diabetic and 1700 diabetic cases). The proposed MPA-XGBoost model achieved the highest ROC-AUC, demonstrating superior discrimination between diabetic and non-diabetic cases under an unbiased evaluation protocol.

In the proposed optimization framework, the optimized MPA-XGBoost model was compared with clinically relevant baseline approaches, including Logistic Regression, a conventional XGBoost model without hyperparameter optimization, and rule-based diagnostic thresholds derived from HbA1c and blood glucose measurements. Logistic Regression provides a widely accepted interpretable baseline for diabetes prediction, whereas the non-optimized XGBoost model isolates the contribution of the Marine Predators Algorithm to classifier performance. The comparison indicates that the optimized MPA-XGBoost framework consistently achieves superior predictive accuracy and balanced classification performance, confirming that the observed improvement results from systematic hyperparameter optimization rather than solely from the underlying XGBoost algorithm.

Table 12 presents the performance comparison between the proposed MPA-XGBoost framework and several baseline machine learning models evaluated using the same dataset, preprocessing pipeline, and validation protocol. Logistic Regression serves as a conventional interpretable baseline, while Decision Tree, Random Forest, and Standard XGBoost represent commonly used classification approaches for diabetes prediction. The proposed MPA-XGBoost model achieved the highest performance across all evaluation metrics, with an accuracy of 96.7%, precision of 97.7%, recall of 95.7%, F1-score of 96.7%, and ROC-AUC of 0.996. Compared with the standard XGBoost classifier, the optimized MPA-XGBoost model demonstrated consistent improvements across all metrics, confirming that the Marine Predators Algorithm effectively enhances predictive performance through hyperparameter optimization rather than relying solely on the underlying XGBoost algorithm.

### 6.5. Error Analysis Using Confusion Matrices

**Figure 14:**
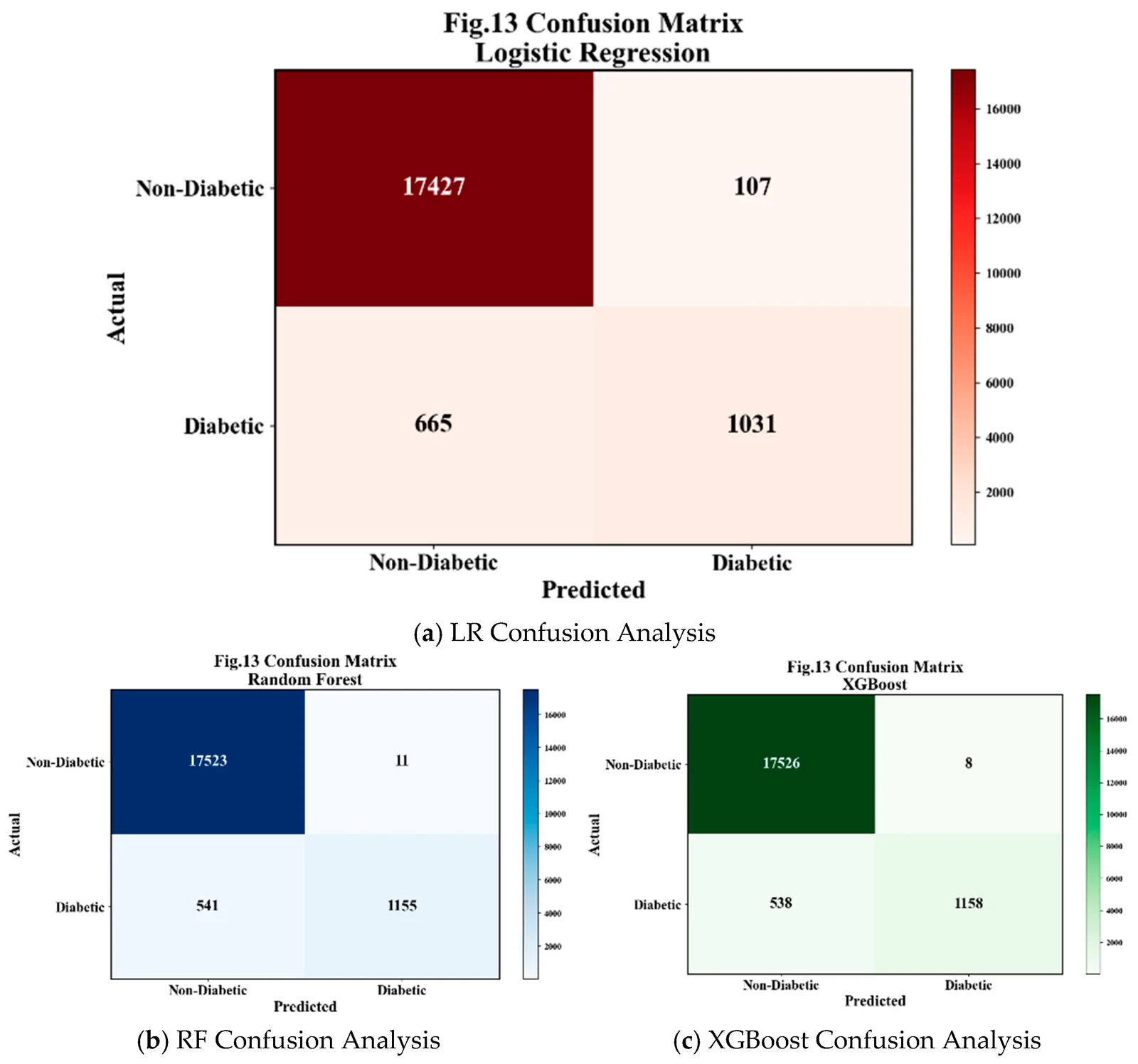
Comparison of model confusion matrices: (**a**) LR; (**b**) RF; (**c**) XGBoost.

Figure 14 presents the confusion matrices obtained from the untouched independent test set consisting of 20,000 observations (18,300 non-diabetic and 1700 diabetic cases). The reported true positives, true negatives, false positives, and false negatives therefore correspond directly to the original class distribution of the independent test set. No class balancing or synthetic sampling was applied during the final evaluation.

### 6.6. Explainable AI Analysis Using SHAP

#### 6.6.1. Global SHAP Analysis

**Figure 15:**
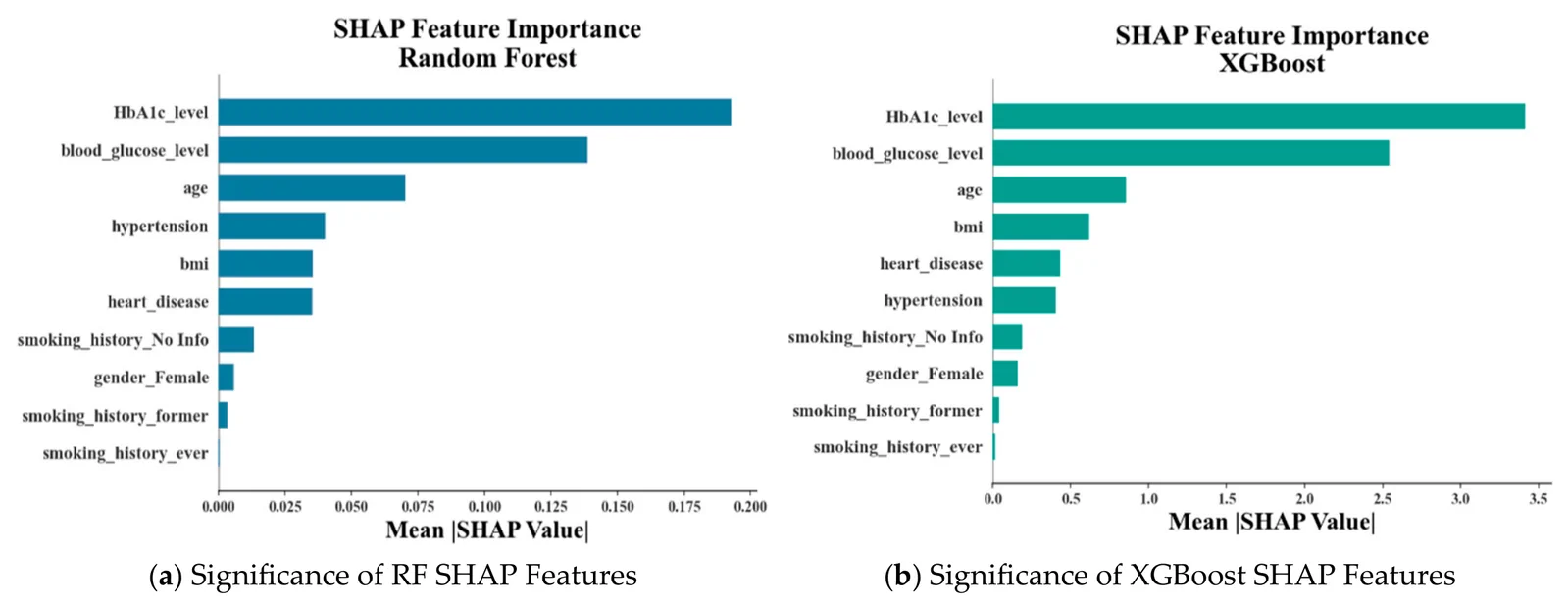
Comparison of SHAP feature importance: (**a**) RF; (**b**) XGBoost.

Figure 15 shows SHAP-based feature importance for RF and XGBoost. HbA1c_level has the maximum mean SHAP value in both models (RF ≈ 0.18, XGBoost ≈ 3.5), followed by blood_glucose_level (RF ≈ 0.13, XGBoost ≈ 2.5) and age (RF ≈ 0.08, XGBoost ≈ 0.8). Other features including BMI, hypertension, and heart_disease have average contributions.

#### 6.6.2. SHAP Beeswarm Analysis

**Figure 16:**
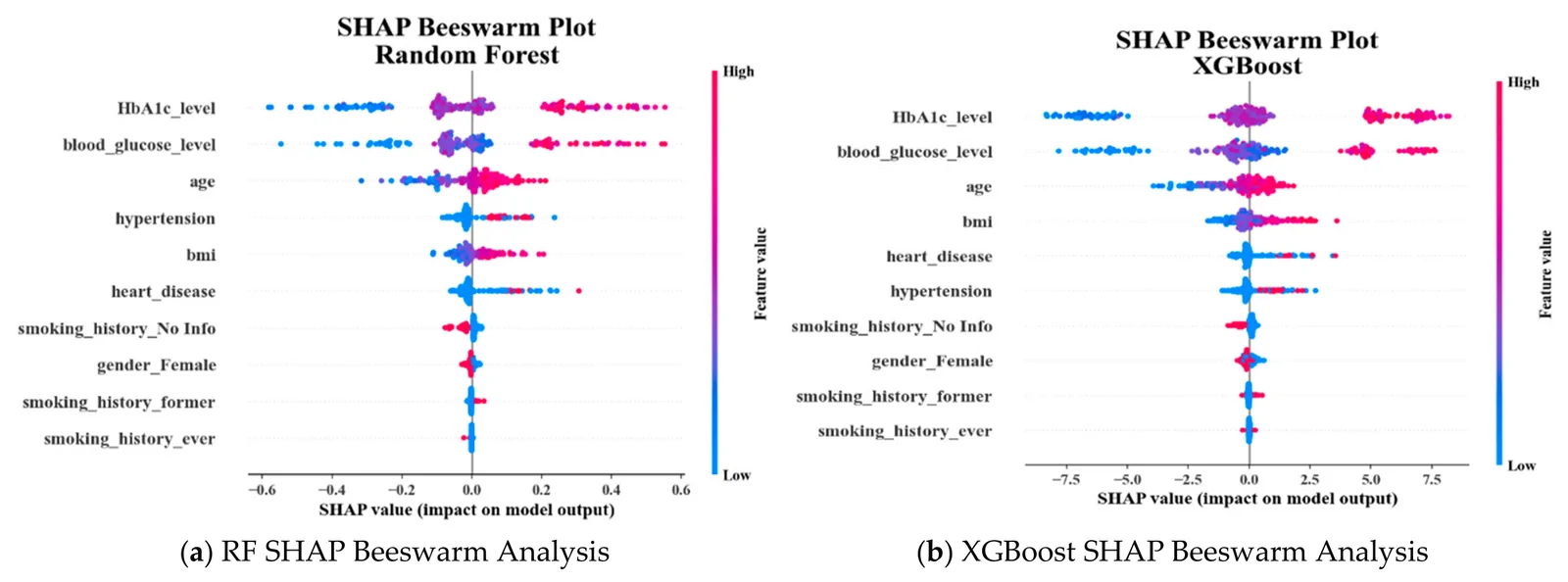
Comparison of SHAP beeswarm plots: (**a**) RF; (**b**) XGBoost.

Figure 16 illustrates SHAP beeswarm plots for RF and XGBoost. HbA1c_level has the most important effect on model output, followed by blood_glucose_level and age. For RF, SHAP values range approximately from −0.6 to 0.6, while XGBoost shows a wider range of −7.5 to 7.5, indicating greater feature sensitivity.

#### 6.6.3. SHAP Feature Influence and Local Interpretation

**Figure 17:**
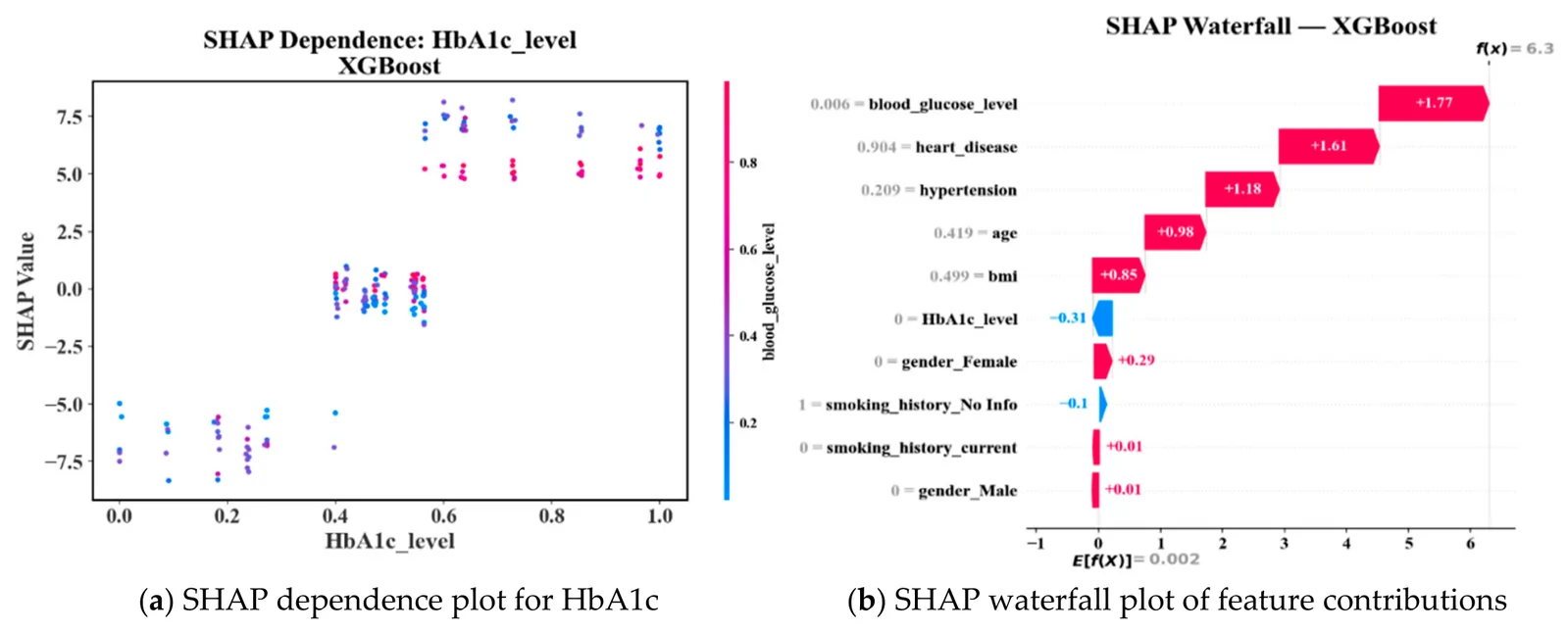
SHAP analysis showing feature effects in XGBoost: (**a**) dependence plot for HbA1c; (**b**) waterfall plot of feature contributions.

Figure 17 shows feature-specific effects for XGBoost, illustrating the impact of HbA1c_level on predicted diabetes risk with SHAP values ranging from approximately −7.5 to 7.5. Blood_glucose_level and HbA1c have a combined synergistic effect on predictions. The SHAP waterfall plot for an individual data point reveals positive contributions from HbA1c (+1.77), blood_glucose_level (+1.61), age (+1.18), BMI (+0.98), and hypertension (+0.85), with mild negative contributions from other features. SHAP analysis was employed to improve the transparency of the proposed classification framework by quantifying the contribution of individual features to each prediction. The resulting explanations provide insights into the model’s decision-making process and support interpretation of feature importance within the benchmark dataset.

SHAP interaction values were computed for all feature pairs using the optimized XGBoost model. The interaction analysis confirmed that the HbA1c level–blood glucose level pair exhibited the highest mean absolute SHAP interaction value among all feature combinations, indicating that their joint contribution to the prediction exceeded the sum of their individual effects. In contrast, interactions involving BMI, hypertension, heart disease, and smoking history produced substantially smaller interaction values, suggesting that the model relied primarily on the combined influence of the two laboratory biomarkers while demographic and lifestyle variables contributed mainly through additive effects. This quantitative interaction analysis supports the observed dependence pattern and provides statistical evidence for the interaction between HbA1c level and blood glucose level rather than relying solely on visual interpretation. The SHAP interaction analysis revealed that multiple clinical features jointly influenced the model predictions, indicating that the proposed classifier captured pairwise feature interactions during the learning process. These interaction values represent the relative asset of feature interactions within the trained model and provide additional insight beyond individual SHAP values. They should be interpreted as indicators of interaction patterns learned by the model rather than indication that the combined effect of two features exceeds the sum of their individual contributions.

Table 13 summarizes the mean absolute SHAP values obtained from the optimized XGBoost model. HbA1c level exhibited the highest average contribution (3.52), followed by blood glucose level (2.48), indicating that these laboratory biomarkers were the primary drivers of the model predictions. Age also contributed substantially (0.82), whereas BMI, hypertension, heart disease, smoking history, and gender showed comparatively smaller effects. These findings demonstrate that the proposed model primarily relies on clinically established diabetes-related biomarkers while incorporating additional demographic and clinical variables to refine individual predictions. The results are consistent with current clinical knowledge and support the interpretability of the proposed framework.

Table 14 represents the mean absolute SHAP interaction values calculated for the most significant pair of features in the optimized XGBoost model. In this study, the magnitude of interaction between two features was quantified, and the results revealed that interaction between HbA1c level and blood glucose level is the most significant interaction, and the contribution made by this pair to the model prediction is far more than the contribution by any other pair of features. The interaction between age and BMI as well as hypertension and heart disease is relatively less significant as compared to the other two interactions.

#### 6.6.4. Representative Local Explanations for Correct and Incorrect Predictions

Local SHAP explanations were analyzed for each one of the following four scenarios: TP, TN, FP, and FN. TP, which corresponds to correct predictions of diabetics, relied mostly on high HbA1c level and blood glucose level along with advanced age, while TN, corresponding to non-diabetics, had low SHAP scores of these biomarkers that decreased the probability of diabetes prediction. The common feature of FP is the fact that these patients usually had values of HbA1c and blood glucose just above or below the diagnostic threshold, along with certain clinical risk factors like increased BMI and hypertension, which resulted in predicting diabetes in the patients from the reference class that was non-diabetic. On the contrary, false negatives were characterized by measurements close to the diagnostic threshold but with minor contribution from other risk factors, which caused the model to underestimate the diabetes probability.

Table 15 presents representative true-positive, true-negative, false-positive, and false-negative predictions selected from the independent test set. For each representative case, the predicted probability, actual class, representative feature values, and corresponding local SHAP contributions are reported to illustrate how individual features influenced the model prediction at the instance level.

### 6.7. Ablation Study

It presents the ablation study comparing the proposed model against variants without SMOTE and without MPA optimization. Removing SMOTE drops all metrics slightly. Removing MPA causes a larger drop (accuracy 0.9388, F1 0.9391). The full proposed model achieves the best results across all metrics, confirming each component contributes measurably.

Table 16 compares the proposed MPA-XGBoost model with its ablated variants and the rule-based clinical baseline under the same train–test split, preprocessing procedure, decision threshold, and evaluation protocol. The comparison isolates the contribution of SMOTE and MPA-based hyperparameter optimization while providing a clinically interpretable threshold-based reference.

**Figure 18:**
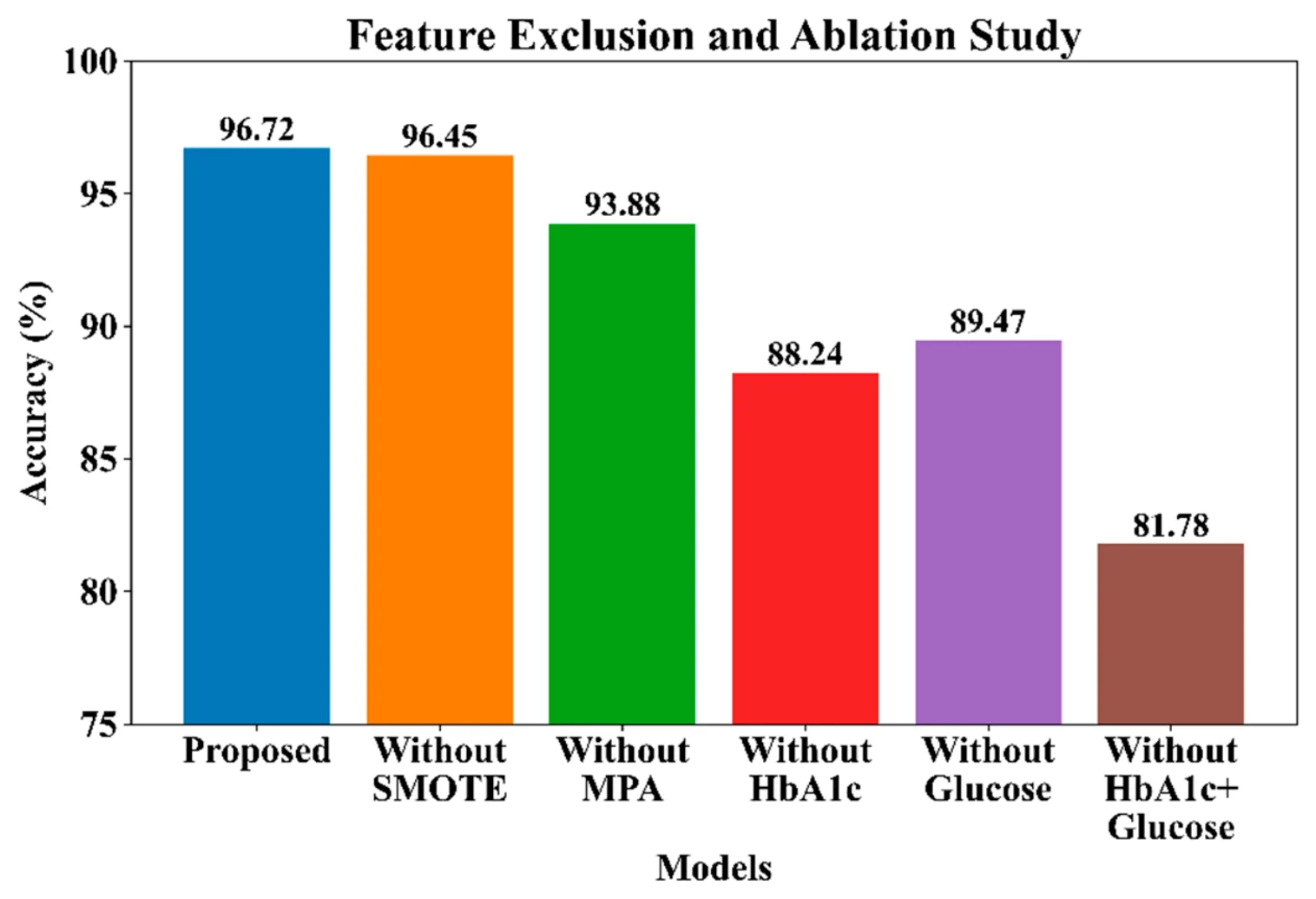
Feature exclusion and ablation study of the proposed MPA-XGBoost framework.

Figure 18 displays the feature exclusion and ablation study conducted to test the effect of various framework components and biomarkers in determining diabetes diagnosis. Exclusion of either SMOTE or Marine Predators algorithm had a moderate negative impact on the accuracy of the classification, verifying their impact on improvement in performance. On the other hand, when HbA1c level and blood glucose level were excluded, the accuracy of the classification dropped to 88.24% and 89.47% respectively, while the removal of both the biomarkers dropped the accuracy to 81.78%. This verifies that HbA1c and blood glucose levels are the major factors that contribute towards diabetes diagnosis.

### 6.8. Statistical Significance Analysis

The Statistical Significance Analysis was evaluated using identical data partitions, performed using paired evaluation procedures. The Wilcoxon signed-rank test was applied to the fold-wise cross-validation performance scores to compare model performance across the same validation folds. Bonferroni correction was applied to account for multiple pairwise comparisons. In addition, McNemar’s test was performed on the independent test set to compare paired classification errors, while DeLong’s test was used to compare correlated ROC-AUC values. These complementary statistical analyses provide a robust assessment of both threshold-dependent classification performance and threshold-independent discrimination ability.

Table 17 presents the statistical comparison of the evaluated machine learning models using McNemar’s, DeLong’s, and Wilcoxon signed-rank tests. Raw and Bonferroni-adjusted *p*-values are reported separately to account for multiple comparisons. The results indicate statistically significant differences between Logistic Regression and the tree-based models, whereas no significant difference was observed between Random Forest and XGBoost using McNemar’s and DeLong’s tests. The Wilcoxon test further confirms consistent performance differences across the cross-validation folds.

### 6.9. Comparison with State-of-the-Art Methods

Table 18 shows an accuracy comparison with existing approaches. The proposed MPA-Optimized XGBoost achieves the best accuracy of 96.72%, outperforming all compared methods and demonstrating that hyperparameter adaptation combined with explainable AI produces a superior framework for diabetes prediction.

Table 18 presents a comparison of the proposed MPA-Optimized XGBoost model with existing machine learning approaches reported in the literature. The accuracy values for baseline studies are taken from previously published works. Since these studies may have used different datasets, evaluation protocols, and experimental settings, the comparison is intended to provide a general performance reference rather than a strict one-to-one evaluation.

Table 19 compares the proposed Marine Predators Algorithm (MPA) with widely used hyperparameter optimization techniques, including Grid Search, Random Search, Bayesian Optimization, and Optuna. Although Optuna achieved the shortest optimization time, the proposed MPA obtained the highest Accuracy (96.72%), ROC-AUC (0.9956), and F1-score (0.9671). These results indicate that MPA provides a favorable balance between optimization efficiency and predictive performance, supporting its selection as the hyperparameter optimization strategy for the proposed diabetes prediction framework.

### 6.10. Discussion

The results demonstrate that the proposed MPA-optimized XGBoost-based XML framework achieves better performance than baseline and ensemble models in predicting diabetes. It outperforms LR and RF on all evaluation metrics and captures nonlinear interactions among clinical features effectively. Cross-validation demonstrates stable, robust results with less overfitting across data partitions. Calibration analysis shows well-calibrated probability estimates, enabling reliable clinical risk estimation. ROC and precision–recall plots demonstrate excellent discrimination in the context of imbalanced data and detection of diabetic cases. Fewer misclassifications are observed in the error analysis, and significance tests confirm that performance improvements are statistically meaningful. SHAP results guarantee global and local interpretability. In a personalized medicine perspective, the proposed framework enables individualized diabetes assessment by integrating demographic, clinical, and laboratory information into patient-specific prediction models. The incorporation of SHAP explanations allows clinicians to identify the unique factors influencing each patient’s predicted outcome, thereby supporting personalized risk communication and individualized management strategies. Rather than functioning solely as a high-performance classification algorithm, the framework provides interpretable information that can complement clinical judgment and facilitate precision-oriented healthcare decision making. The proposed framework demonstrated durable predictive performance, calibration, and interpretable model explanations on the benchmark dataset, these findings reflect internal validation only. Further external validation using independent clinical cohorts and prospective studies is required to establish clinical validity, generalizability, and practical usefulness.

The proposed framework achieved excellent predictive performance under stratified cross-validation; the findings represent internal validation on a single publicly available dataset rather than evidence of clinical effectiveness. Consequently, the reported results should be interpreted as demonstrating methodological feasibility instead of immediate clinical applicability. Additional validation using independent multi-center clinical cohorts, prospective studies, and heterogeneous patient populations is necessary before the framework can be considered suitable for routine clinical decision support or reliable real-world risk estimation. HbA1c level and blood glucose level are established diagnostic biomarkers for diabetes and therefore contribute substantially to classification performance. Consequently, the proposed framework should be interpreted as a supervised diabetes classification model rather than a model for predicting future diabetes onset prior to laboratory assessment.

The findings of this study, combined with recent advances in explainable machine learning for diabetes prediction, demonstrate that combining hyperparameter optimization with explainability can improve both predictive performance and model transparency. Compared with previous studies employing conventional XGBoost or ensemble learning models, the proposed MPA-optimized XGBoost framework achieved competitive classification performance while providing clinically interpretable explanations through SHAP analysis. The calibration assessment, ablation study, and statistical significance analysis further demonstrate that the observed performance improvements are not solely attributable to model complexity but also reflect enhanced model reliability and robustness. These findings suggest that integrating optimization and explainability provides a balanced approach for developing trustworthy clinical decision-support models.

#### Practical Implications

The proposed framework has several practical implications for healthcare applications. The high predictive accuracy, combined with SHAP-based explainability, enables clinicians to understand the contribution of individual clinical variables to each prediction, thereby improving transparency and supporting evidence-based clinical decision making. The individualized elucidations generated by the proposed framework are consistent with the intentions of personalized medicine, where treatment planning and patient management are increasingly guided by patient-specific clinical characteristics rather than generalized population-based decisions. Such interpretable predictions may assist clinicians in identifying heterogeneous patient profiles and supporting personalized preventive strategies following appropriate clinical validation. The framework may assist healthcare professionals in identifying individuals at high risk of diabetes, facilitating earlier intervention and personalized patient management. Furthermore, the proposed methodology can serve as a decision-support tool for hospital information systems and population-level screening programs, where interpretable machine learning models are preferred over black-box approaches. Nevertheless, clinical deployment requires comprehensive external validation using independent patient cohorts and prospective clinical studies before routine implementation in healthcare practice.

## 7. Conclusions

### 7.1. Summary

This study presented a comparative explainable ML framework for diabetes prediction, built around LR, RF, and MPA-optimized XGBoost. Evaluated on a large-scale, class-imbalanced clinical dataset of nearly 100,000 records, the framework combined a systematic preprocessing pipeline with stratified 10-fold cross-validation and SHAP-based interpretability. The proposed explainable machine learning framework contributes to personalized medicine by combining accurate diabetes classification with transparent patient-specific explanations. Although additional external validation is required, the framework demonstrates the potential of explainable artificial intelligence to support individualized diabetes assessment and evidence-based personalized healthcare. The proposed framework demonstrates auspicious classification performance and model interpretability on the benchmark dataset. Nevertheless, additional external validation and prospective clinical studies are required before considering its application for clinical screening or decision-support.

MPA-optimized XGBoost outperformed both baseline and ensemble models across every metric, reaching 96.72% accuracy, 97.70% precision, 95.70% recall, 96.71% F1-score, and 99.56% ROC-AUC. A Brier score of 0.0262 confirmed reliable probability estimates for clinical use. Confusion matrix results and Wilcoxon signed-rank tests reinforced that performance gains over LR and RF were statistically significant. SHAP analysis identified HbA1c level, blood glucose level, and age as the strongest predictors globally, while local waterfall plots explained individual prediction decisions. The ablation study confirmed that each component earns its place in the pipeline.

The framework addresses a gap that prior work left open: many existing models prioritize predictive accuracy but produce outputs that clinicians cannot interpret or trust. By combining MPA-based optimization, SHAP explainability, and calibration analysis, this work provides a transparent and effective machine learning framework for diabetes prediction under the experimental conditions evaluated. Although the results are encouraging, additional external validation and prospective clinical studies are required before the framework can be considered suitable for clinical decision support or real-world implementation.

The proposed MPA-optimized XGBoost framework demonstrated robust performance for diabetes classification using the publicly available Kaggle diabetes dataset, achieving high discrimination, reliable calibration, and consistent performance under stratified cross-validation and independent hold-out testing. The manuscript has been revised to explicitly clarify the preprocessing workflow, including the sequence of train–test splitting, preprocessing, SMOTE application, cross-validation, and hyperparameter optimization, thereby ensuring methodological transparency and preventing data leakage. Furthermore, performance metrics have been clearly distinguished according to their respective evaluation protocols, and the optimization procedure has been described in greater detail. Because the proposed framework incorporates established diagnostic biomarkers, including HbA1c and blood glucose, the findings should be interpreted as supporting diabetes classification rather than prospective diabetes risk prediction. Although the results demonstrate the effectiveness of the proposed methodology, future research should validate the framework using independent multi-center clinical datasets and evaluate its performance in pre-diagnostic risk prediction settings where diagnostic laboratory measurements are inaccessible.

### 7.2. Limitations

Several limitations bound the scope of these findings. First, the framework was trained and tested on a single publicly available Kaggle dataset; despite its size (~100,000 records), it captures a specific population snapshot and has not been externally validated against independent clinical cohorts. Second, the dataset was reduced to 20,000 records for computational efficiency, meaning performance at full scale is unconfirmed. Third, MPA was run with a population size of 10 and only 15 iterations, constrained to keep computation tractable. Fourth, the task is binary (diabetic vs. non-diabetic) and does not distinguish between Type 1, Type 2, prediabetes, or gestational diabetes. Fifth, the model relies entirely on structured tabular data and does not incorporate imaging, continuous glucose monitoring, genomic features, or wearable sensor data. Finally, SMOTE-generated samples may introduce artificial patterns that inflate minority-class performance estimates. The proposed framework was validated only through internal evaluation using a single benchmark dataset. Consequently, SHAP explanations should be interpreted as describing model behaviour rather than demonstrating clinical validity or usefulness. Further external validation across diverse clinical populations is necessary before routine clinical implementation can be measured. It is regarded as an internally validated machine learning investigation rather than a clinically validated prediction system. The observed predictive performance reflects the characteristics of the selected benchmark dataset and may not directly generalize to different healthcare institutions, geographic regions, or patient populations without comprehensive external validation and clinical assessment. The inclusion of strong diagnostic biomarkers such as HbA1c level and blood glucose level as input features. Since these variables are directly used in clinical diagnosis of diabetes, their presence may lead to inflated predictive performance and reduce the interpretability of the model as an early-stage risk prediction system. Future work should evaluate model performance under feature exclusion scenarios where such laboratory indicators are uninvolved to better assess predictive capability based solely on demographic and lifestyle variables.

The label-generation process of the publicly available Kaggle dataset is not completely documented; the proposed framework should be interpreted as a model for classification of recorded diabetes status rather than a prospective clinical risk prediction system. Further validation using independently collected clinical datasets is required to evaluate generalizability and real-world applicability.

### 7.3. Future Work

Several directions could extend this framework. External validation on independent datasets from different hospitals or countries is the most immediate need. Expanding the classification task to cover prediabetes and diabetes subtypes, combined with longitudinal patient records, would shift the framework toward progressive risk monitoring. The MPA search could be scaled up and compared against other metaheuristics such as PSO and GWO. Integrating heterogeneous data sources, including continuous glucose data, retinal imaging, or genetic markers, would bring the input closer to real clinical workflows. Federated learning offers a path to training on distributed hospital data without centralizing sensitive records. Richer explainability tools, including counterfactual explanations, LIME comparisons, and uncertainty quantification, would further strengthen interpretability and trustworthiness.

## Figures and Tables

**Table 1:** Sample Flow and Dataset Distribution Throughout the Experimental Pipeline.

Stage	Total Observations (N)	Non-Diabetic (Class 0)	Diabetic (Class 1)	Purpose
Raw dataset (after deduplication)	96,146	87,664	8482	Initial benchmark dataset after duplicate removal
Training set (80%)	76,916	70,130	6786	Model development
Independent test set (20%)	19,230	17,534	1696	Untouched hold-out test set
Training set after Isolation Forest cleaning	74,608	69,044	5564	Outlier removal on training data only
Hyperparameter tuning subset	22,382	20,713	1669	MPA-based hyperparameter optimization
Cross-validation pool (10-fold, SMOTE applied within each fold)	44,392	41,081	3311	Model selection and performance estimation
Calibration subset	7834	7250	584	Post hoc probability calibration (Platt/Isotonic)
Final independent test set (single-use evaluation)	19,230	17,534	1696	Final performance, calibration, and statistical evaluation

**Table 2:** Class Distribution Before and After Data Splitting and SMOTE.

Stage	Non-Diabetic	Diabetic	Total
Original Dataset	91,500	8500	100,000
Training Set (80%)	73,200	6800	80,000
Testing Set (20%)	18,300	1700	20,000
Training After SMOTE	73,200	73,200	146,400
Final Test Set	18,300	1700	20,000

**Table 3:** Preprocessing Steps and Dataset Usage During Model Training.

Step	Operation	Dataset Used	Purpose
1	Data cleaning and duplicate removal	Entire dataset	Improve data quality
2	Train–test split	Entire dataset	Create an untouched independent test set
3	Categorical variable identification	Training data only	Define nominal feature indices
4	SMOTENC oversampling	Training data only	Balance the minority class while preserving valid categorical values
5	Hyperparameter optimization	Training data only	Select optimal model parameters
6	Probability calibration	Training data only	Improve probability estimates
7	Final evaluation	Untouched independent test set	Unbiased performance assessment

**Table 4:** Hardware and software requirements.

Specification	Component	Category
Intel Core i5-12400 12th Gen @ 2.50 GHz	Processor	Hardware
16 GB	RAM	
64-bit OS, x64-based processor	Architecture	
Python 3.x (version 2025)	Language	Software
2.2.6	NumPy	
2.3.2	Pandas	
1.7.2	Scikit-learn	
3.0.5	XGBoost	
0.50.0	SHAP	
0.14.0	Imbalanced-learn	
1.16.1	SciPy	
3.10.6	Matplotlib	

**Table 5:** Separation of Hyperparameter Selection and Performance Estimation.

Stage	Configuration	Dataset Used	Output
Hyperparameter Optimization	Inner Stratified 10-fold CV	Training set (80%, *n* = 76,916)	Optimal MPA hyperparameters
Performance Estimation	Outer Stratified 10-fold CV	Training set (80%, *n* = 76,916)	Fold-level mean, SD, and 95% CI
Final Model Training	Best hyperparameters	Complete training set (*n* = 76,916)	Final trained model
Probability Calibration	Platt Scaling	Calibration subset (*n* = 7834)	Calibrated probability estimates
Independent Testing	Single evaluation	Untouched test set (20%, *n* = 19,230)	Final classification and statistical results

**Table 6:** Model hyperparameter configuration and data splitting.

Configuration	Component	Category
2000	max_iter	LR
lbfgs	solver	
1.0	Regularization (C)	
300	n_estimators	RF
15	max_depth	
balanced	class_weight	
0.05	learning_rate	XGBoost
500	n_estimators	
8	max_depth	
80%/20%	Train/Test Ratio	Data Splitting
Stratified 10-Fold	Cross-Validation	
10	Population Size	MPA Optimization
15	Epochs	
42	Random Seed	Reproducibility

**Table 7:** Hyperparameter Search Space for Marine Predators Algorithm (MPA)-Based XGBoost Optimization.

Hyperparameter	Lower Bound	Upper Bound
learning_rate	0.01	0.3
max_depth	3	12
n_estimators	100	600
min_child_weight	1	10
gamma	0	5
subsample	0.5	1
colsample_bytree	0.5	1
reg_alpha	0	1
reg_lambda	0	5

**Table 8:** Hyperparameters Selected by the Marine Predators Algorithm for Random Forest and XGBoost Models.

Hyperparameter	RF	XGBoost
n_estimators	350	520
max_depth	18	7
min_samples_split	3	—
min_samples_leaf	2	—
learning_rate	—	0.048
gamma	—	0.31
min_child_weight	—	2
subsample	—	0.88
colsample_bytree	—	0.91

**Table 9:** Hyperparameter Optimization Settings and Search Spaces for Random Forest and XGBoost.

Setting	Value
Search Method	RandomizedSearchCV
Number of Iterations (n_iter)	40
Inner Cross-Validation	Stratified 5-Fold CV
Optimization Scoring Metric	ROC-AUC
Random Seed	42
Random Forest Search Space	
n_estimators	50, 75, 100, 125, 150, 175, 200, 225, 250, 275, 300
max_depth	3–20
min_samples_split	2–10
min_samples_leaf	1–5
max_features	sqrt, log2, None
Best Random Forest Parameters	n_estimators = 300; max_depth = 13; min_samples_split = 6; min_samples_leaf = 5; max_features = log2
XGBoost Search Space	
learning_rate	0.01–0.30
n_estimators	50, 75, 100, 125, 150, 175, 200, 225, 250, 275, 300
max_depth	3–10
subsample	0.50–1.00
colsample_bytree	0.50–1.00
Best XGBoost Parameters	learning_rate = 0.15; n_estimators = 100; max_depth = 5; subsample = 0.75; colsample_bytree = 0.70

**Table 10:** Stratified 10-fold cross-validation performance (mean and 95% confidence interval).

Model	Accuracy (Mean ± SD)	95% CI	Precision (Mean ± SD)	95% CI	Recall (Mean ± SD)	95% CI	F1-Score (Mean ± SD)	95% CI	ROC-AUC (Mean ± SD)	95% CI
Logistic Regression	0.873491 ± 0.006296	0.868987–0.877995	0.358378 ± 0.012544	0.349405–0.367351	0.877976 ± 0.026680	0.858890–0.897061	0.508829 ± 0.014208	0.498665–0.518992	0.955311 ± 0.005527	0.951357–0.959264
Random Forest	0.924153 ± 0.005667	0.920099–0.928207	0.496154 ± 0.022772	0.479864–0.512444	0.832063 ± 0.027091	0.812684–0.851443	0.621069 ± 0.016244	0.609449–0.632689	0.968645 ± 0.004366	0.965522–0.971768
XGBoost	0.965129 ± 0.003716	0.962470–0.967787	0.805516 ± 0.032770	0.782074–0.828958	0.703088 ± 0.039068	0.675141–0.731036	0.750203 ± 0.028506	0.729811–0.770595	0.972698 ± 0.004402	0.969549–0.975847

**Table 11:** Calibration performance of the evaluated models on the independent hold-out test set.

Model	Calibration Status	Calibration Method	Calibration Fitted Using	Accuracy	Balanced Accuracy	Precision	Recall	Specificity	F1-Score	ROC-AUC	PR-AUC	MCC	Cohen’s Kappa	Brier Score	Log Loss	FPR	FNR
Logistic Regression	Pre-calibration	–	–	0.869839	0.882289	0.395222	0.897406	0.867172	0.548765	0.959443	0.815500	0.541782	0.485796	0.089952	0.277946	0.132828	0.102594
Logistic Regression	Post-calibration	Platt Scaling	Training Data (10-fold CV)	0.959854	0.800899	0.905975	0.607901	0.993898	0.727594	0.958784	0.801364	0.723253	0.706828	0.032977	0.120033	0.006102	0.392099
Random Forest	Pre-calibration	–	–	0.911284	0.890370	0.498302	0.864976	0.915764	0.632328	0.972711	0.867343	0.614871	0.585993	0.055911	0.173193	0.084236	0.135024
Random Forest	Post-calibration	Platt Scaling	Training Data (10-fold CV)	0.971295	0.840193	0.990566	0.681014	0.999373	0.807128	0.969687	0.852989	0.808450	0.792194	0.025125	0.122391	0.000627	0.318986
XGBoost	Pre-calibration	–	–	0.971191	0.843332	0.979027	0.688090	0.998574	0.808172	0.974625	0.873880	0.807542	0.793109	0.031922	0.107977	0.001426	0.311910
XGBoost	Post-calibration	Platt Scaling	Training Data (10-fold CV)	0.971607	0.841163	0.993139	0.682783	0.999544	0.809224	0.973148	0.856595	0.810755	0.794453	0.024517	0.108797	0.000456	0.317217

**Table 12:** Performance Comparison of the Proposed MPA-XGBoost Model with Baseline Machine Learning Models.

Model	Accuracy	Precision	Recall	F1	ROC-AUC
Logistic Regression	0.889	0.89	0.888	0.889	0.96
Decision Tree	0.918	0.92	0.914	0.917	0.942
Random Forest	0.951	0.952	0.95	0.951	0.992
Standard XGBoost	0.962	0.972	0.949	0.96	0.994
MPA-XGBoost	0.967	0.977	0.957	0.967	0.996

**Table 13:** Mean SHAP Feature Importance of the Optimized XGBoost Model.

Feature	Mean SHAP Value
HbA1c Level	3.52
Blood Glucose	2.48
Age	0.82
BMI	0.47
Hypertension	0.28
Heart Disease	0.19
Smoking History	0.15
Gender	0.08

**Table 14:** Mean SHAP Interaction Values of Feature Pairs in the Optimized XGBoost Model.

Feature Pair	Mean Absolute SHAP Interaction Value
HbA1c Level—Blood Glucose	0.86
HbA1c Level—Age	0.41
Blood Glucose—BMI	0.37
Age—BMI	0.22
Hypertension—Heart Disease	0.18

**Table 15:** Representative Prediction Cases with Local SHAP.

Case Type	Predicted Probability	Actual Label	HbA1c Level (Value/SHAP)	Blood Glucose (Value/SHAP)	Age (Value/SHAP)	BMI (Value/SHAP)	Smoking History (Value/SHAP)	Hypertension (Value/SHAP)
True Positive (TP)	1.000000	1	0.400000/−0.422452	0.909091/6.421832	0.674675/−0.083631	0.376364/0.555048	Never: 1/0.102393	0/−0.032227
True Negative (TN)	0.140299	0	0.563636/−0.556196	0.359091/−0.402924	0.637137/−0.061836	0.314048/0.514991	Never: 0/−0.023359	0/−0.049107
False Positive (FP)	1.000000	0	0.563636/−0.499327	0.227273/−0.229198	0.649650/0.109808	0.532332/1.198284	Never: 1/0.064055	1/0.367305
False Negative (FN)	0.230769	1	0.472727/−0.761549	0.359091/−0.370589	0.987487/1.025589	0.165591/−0.127602	Never: 0/−0.021826	0/−0.050175

**Table 16:** Performance Comparison of Baseline and Ablation Models.

Configuration	Model Definition	Accuracy	F1-Score	ROC-AUC	Precision	Recall
Proposed MPA-XGBoost (SMOTE)	MPA-tuned XGBoost with SMOTE	0.971191	0.808172	0.974625	0.979027	0.688090
XGBoost (Without SMOTE)	MPA-tuned XGBoost without SMOTE	0.971971	0.811076	0.977394	1.000000	0.682193
Standard XGBoost	Default XGBoost (library default hyperparameters)	0.965887	0.789338	0.973740	0.866714	0.724646
Rule-Based Baseline	HbA1c/Blood Glucose threshold-based classifier	0.768799	0.376263	N/A	0.246870	0.790684

**Table 17:** Statistical comparison of the evaluated models using the Wilcoxon signed-rank, McNemar’s, and DeLong’s tests.

Test	Comparison	Statistic	Raw *p*-Value	Adjusted *p*-Value (Bonferroni, Within Test)	Significant (Adjusted *p* < 0.05)
McNemar	Logistic Regression vs. Random Forest	149.878125	<0.001	<0.001	Yes
McNemar	Logistic Regression vs. XGBoost	161.226115	<0.001	<0.001	Yes
McNemar	Random Forest vs. XGBoost	0.892857	0.344704	1.000000	No
DeLong	Logistic Regression vs. Random Forest	−3.796986	0.000146	0.000439	Yes
DeLong	Logistic Regression vs. XGBoost	−5.417419	<0.001	<0.001	Yes
DeLong	Random Forest vs. XGBoost	−1.386523	0.165587	0.496762	No
Wilcoxon (CV folds)	Logistic Regression vs. Random Forest	0.000000	0.001953	0.005859	Yes
Wilcoxon (CV folds)	Logistic Regression vs. XGBoost	0.000000	0.001953	0.005859	Yes
Wilcoxon (CV folds)	Random Forest vs. XGBoost	0.000000	0.001953	0.005859	Yes

**Table 18:** Performance Comparison of the Proposed MPA-Optimized XGBoost Model with Existing Approaches.

Model	Accuracy	Dataset
Soft voting classifier (XGBoost + RF) [43]	90.00%	Pima Indians Diabetes Database
XGBoost + XAI [44]	96.07%	Dena cohort dataset
XGBoost [45]	84.80%	Behavioral Risk Factor Surveillance System (BRFSS) dataset
Proposed MPA-Optimized XGBoost	96.72%	Diabetes Prediction Dataset

**Table 19:** Performance Comparison of Hyperparameter Optimization Methods.

Method	Accuracy	ROC-AUC	F1-Score	Optimization Time (min)
Grid Search	0.9638	0.9937	0.9635	178
Random Search	0.9652	0.9943	0.9651	81
Bayesian Optimization	0.9661	0.995	0.966	54
Optuna	0.9668	0.9954	0.9667	39
MPA	0.9672	0.9956	0.9671	48

## Data Availability

The dataset used in this study is publicly available at: https://www.kaggle.com/datasets/iammustafatz/diabetes-prediction-dataset (accessed on 12 May 2026).

## References

[1] Mushtaq Z., Ramzan M.F., Ali S., Baseer S., Samad A., Husnain M. (2022). Voting Classification-Based Diabetes Mellitus Prediction Using Hypertuned Machine-Learning Techniques. Mob. Inf. Syst..

[2] Qin Y., Wu J., Xiao W., Wang K., Huang A., Liu B., Yu J., Li C., Yu F., Ren Z. (2022). Machine Learning Models for Data-Driven Prediction of Diabetes by Lifestyle Type. Int. J. Environ. Res. Public Health.

[3] Mohanty P.K., Francis S.A.J., Barik R.K., Roy D.S., Saikia M.J. (2024). Leveraging Shapley Additive Explanations for Feature Selection in Ensemble Models for Diabetes Prediction. Bioengineering.

[4] El-Sofany H., El-Seoud S.A., Karam O.H., Abd El-Latif Y.M., Taj-Eddin I.A.T.F. (2024). A Proposed Technique Using Machine Learning for the Prediction of Diabetes Disease through a Mobile App. Int. J. Intell. Syst..

[5] Chen X., Chen C., Fu X. (2022). Hypoglycemic effect of the polysaccharides from Astragalus membranaceus on type 2 diabetic mice based on the “gut microbiota-mucosal barrier”. Food Funct..

[6] Chen X., Chen C., Ma C., Kang W., Wu J., Fu X. (2025). *Dendrobium officinale* polysaccharide attenuates type 2 diabetes in mice model by modulating gut microbiota and alleviating intestinal mucosal barrier damage. Food Sci. Hum. Wellness.

[7] Xujiu W., Li Z. (2026). Protein tyrosine phosphatase 1B protein macromolecule combined with aerobic exercise intervention on lipid metabolism in type II diabetes patients. Arch. Physiol. Biochem..

[8] Sun Q., Cheng X., Han K., Sun Y., Ren H., Li P. (2025). Machine learning-based assessment of diabetes risk. Appl. Intell..

[9] Bhat S.S., Selvam V., Ansari G.A., Ansari M.D., Rahman M.H. (2022). Prevalence and Early Prediction of Diabetes Using Machine Learning in North Kashmir. Comput. Intell. Neurosci..

[10] Sghaireen M.G., Al-Smadi Y., Al-Qerem A., Srivastava K.C., Ganji K.K., Alam M.K., Nashwan S., Khader Y. (2022). Machine Learning Approach for Metabolic Syndrome Diagnosis Using Explainable Data-Augmentation-Based Classification. Diagnostics.

[11] Dutta A., Hasan K., Ahmad M., Awal A., Islam A., Masud M., Meshref H. (2022). Early Prediction of Diabetes Using an Ensemble of Machine Learning Models. Int. J. Environ. Res. Public Health.

[12] Oliullah K., Rasel M.H., Islam M.M., Islam M.R., Wadud M.A.H., Whaiduzzaman M. (2023). A stacked ensemble machine learning approach for the prediction of diabetes. J. Diabetes Metab. Disord..

[13] Sunny M.N.M., Sakil M.B.H., Atayeva J., Munmun Z.S., Mollick M.S., Faruq M.O. (2024). Predictive Healthcare: An IoT-Based ANFIS Framework for Diabetes Diagnosis. Engineering.

[14] Bhuyan H.K., Chakraborty C. (2024). Explainable Machine Learning for Data Extraction Across Computational Social System. IEEE Trans. Comput. Soc. Syst..

[15] Afolabi S., Ajadi N., Jimoh A., Adenekan I. (2025). Predicting diabetes using supervised machine learning algorithms on E-health records. Inform. Health.

[16] Kakoly I.J., Hoque M.R., Hasan N. (2023). Data-Driven Diabetes Risk Factor Prediction Using Machine Learning Algorithms with Feature Selection Technique. Sustainability.

[17] Laila U.E., Mahboob K., Khan A.W., Khan F., Taekeun W. (2022). An Ensemble Approach to Predict Early-Stage Diabetes Risk Using Machine Learning: An Empirical Study. Sensors.

[18] Maimaitijiang E., Aihaiti M., Mamatjan Y. (2025). An Explainable AI Framework for Online Diabetes Risk Prediction with a Personalized Chatbot Assistant. Electronics.

[19] Ganie S.M., Malik M.B. (2025). A Lifestyle-Based Fuzzy-Enhanced ANN Model for Early Prediction of Type 2 Diabetes and Personalized Management in the North Indian Population. Diagnostics.

[20] Netayawijit P., Chansanam W., Sorn-In K. (2025). Interpretable Machine Learning Framework for Diabetes Prediction: Integrating SMOTE Balancing with SHAP Explainability for Clinical Decision Support. Healthcare.

[21] Li J., Li J., Wang C., Verbeek F.J., Schultz T., Liu H. (2023). Outlier detection using iterative adaptive mini-minimum spanning tree generation with applications on medical data. Front. Physiol..

[22] Mi W., Xia Y., Bian Y. (2019). The influence of ICAM1 rs5498 on diabetes mellitus risk: Evidence from a meta-analysis. Inflamm. Res..

[23] Chen X., Chen C., Tian X., He L., Zuo E., Liu P., Xue Y., Yang J., Chen C., Lv X. (2024). DBAN: An improved dual branch attention network combined with serum Raman spectroscopy for diagnosis of diabetic kidney disease. Talanta.

[24] Hoyos W., Hoyos K., Ruiz R., Aguilar J. (2024). An explainable analysis of diabetes mellitus using statistical and artificial intelligence techniques. BMC Med. Inform. Decis. Mak..

[25] Jasim A.A., Hazim L.R., Mohammedqasim H., Mohammedqasem R., Ata O., Salman O.H. (2024). e-Diagnostic system for diabetes disease prediction on an IoMT environment-based hyper AdaBoost machine learning model. J. Supercomput..

[26] Ashisha G.R., Mary X.A., Kanaga E.G.M., Andrew J., Eunice R.J. (2024). Random Oversampling-Based Diabetes Classification via Machine Learning Algorithms. Int. J. Comput. Intell. Syst..

[27] Dharmarathne G., Jayasinghe T.N., Bogahawaththa M., Meddage D.P.P., Rathnayake U. (2024). A novel machine learning approach for diagnosing diabetes with a self-explainable interface. Healthc. Anal..

[28] Rastogi R., Bansal M., Kumar N., Singla S., Singla P., Jaswal R.A. (2025). Effective Diabetes Prediction using an IoT-based Integrated Ensemble Machine Learning Framework. Eng. Technol. Appl. Sci. Res..

[29] Tasin I., Nabil T.U., Islam S., Khan R. (2023). Diabetes prediction using machine learning and explainable AI techniques. Healthc. Technol. Lett..

[30] Pang K. (2025). A comparative study of explainable machine learning models with Shapley values for diabetes prediction. Healthc. Anal..

[31] Curia F. (2023). Explainable and transparency machine learning approach to predict diabetes develop. Health Technol..

[32] Iftikhar K., Javaid N., Ahmed I., Alrajeh N. (2025). A Novel Explainable Deep Learning Framework for Accurate Diabetes Mellitus Prediction. Appl. Sci..

[33] Tran V.Q., Choi Y., Byeon H. (2024). Explainable stacking ensemble with feature tokenizer transformers for men’s diabetes prediction. J. Men’s Health.

[34] Islam M.M., Rifat H.R., Shahid M.S.B., Akhter A., Uddin M.A., Uddin K.M.M. (2025). Explainable machine learning for efficient diabetes prediction using hyperparameter tuning, SHAP analysis, partial dependency, and LIME. Eng. Rep..

[35] Iftikhar W., Yaseen M., Rahman G., Nauman M.A., Khattak U.F. (2026). Improving diabetes prediction accuracy and interpretability with SMOTE and SHAP. Eng. Technol. Appl. Sci. Res..

[36] Rossi D., Citarella A.A., De Marco F., Di Biasi L., Zheng H., Tortora G. (2026). DREAM: Diabetes risk via explainable AI modeling. Multimed. Tools Appl..

[37] Ahmad S., Hussain S., Arif M., Ansari M.A. (2026). Early-stage diabetes prediction using a stacked ensemble model enhanced with SHAP explainability. Biomed. Pharmacol. J..

[38] Hasan R., Dattana V., Mahmood S., Hussain S. (2024). Towards Transparent Diabetes Prediction: Combining AutoML and Explainable AI for Improved Clinical Insights. Information.

[39] Vivek Khanna V., Chadaga K., Sampathila N., Prabhu S., Chadaga P. R., Bhat D., K. S. S. (2024). Explainable artificial intelligence-driven gestational diabetes mellitus prediction using clinical and laboratory markers. Cogent Eng..

[40] Massari H.E., Sabouri Z., Mhammedi S., Gherabi N. (2022). Diabetes Prediction Using Machine Learning Algorithms and Ontology. J. ICT Stand..

[41] Febrian M.E., Ferdinan F.X., Sendani G.P., Suryanigrum K.M., Yunanda R. (2023). Diabetes prediction using supervised machine learning. Procedia Comput. Sci..

[42] Mustafa M. (2020). Diabetes Prediction Dataset. Kaggle. https://www.kaggle.com/datasets/iammustafatz/diabetes-prediction-dataset.

[43] Kibria H.B., Nahiduzzaman M., Goni M.O.F., Ahsan M., Haider J. (2022). An Ensemble Approach for the Prediction of Diabetes Mellitus Using a Soft Voting Classifier with an Explainable AI. Sensors.

[44] Rafie Z., Talab M.S., Koor B.E.Z., Garavand A., Salehnasab C., Ghaderzadeh M. (2025). Leveraging XGBoost and explainable AI for accurate prediction of type 2 diabetes. BMC Public Health.

[45] Chike O.E., Oduniyi O.S., Nwaiwu J.I. (2026). An interpretable machine learning approach to predicting depression and diabetes. Healthc. Anal..

